# Molecular signatures in prion disease: altered death receptor pathways in a mouse model

**DOI:** 10.1186/s12967-024-05121-x

**Published:** 2024-05-27

**Authors:** Ranjit Kumar Giri

**Affiliations:** Molecular and Cellular Neuroscience Division, National Brain Research Centre, Manesar, Gurgaon, Haryana 122052 India

**Keywords:** Prion disease mouse model, Neuropathology, DR3, DR5, TL1A, TRAIL, TRADD, FADD, TRAF2, RIPK1

## Abstract

**Background:**

Prion diseases are transmissible and fatal neurodegenerative diseases characterized by accumulation of misfolded prion protein isoform (PrP^Sc^), astrocytosis, microgliosis, spongiosis, and neurodegeneration. Elevated levels of cell membrane associated PrP^Sc^ protein and inflammatory cytokines hint towards the activation of death receptor (DR) pathway/s in prion diseases. Activation of DRs regulate, either cell survival or apoptosis, autophagy and necroptosis based on the adaptors they interact. Very little is known about the DR pathways activation in prion disease. DR3 and DR5 that are expressed in normal mouse brain were never studied in prion disease, so also their ligands and any DR adaptors. This research gap is notable and investigated in the present study.

**Methods:**

C57BL/6J mice were infected with Rocky Mountain Laboratory scrapie mouse prion strain. The progression of prion disease was examined by observing morphological and behavioural abnormalities. The levels of PrP isoforms and GFAP were measured as the marker of PrP^Sc^ accumulation and astrocytosis respectively using antibody-based techniques that detect proteins on blot and brain section. The levels of DRs, their glycosylation and ectodomain shedding, and associated factors warrant their examination at protein level, hence western blot analysis was employed in this study.

**Results:**

Prion-infected mice developed motor deficits and neuropathology like PrP^Sc^ accumulation and astrocytosis similar to other prion diseases. Results from this research show higher expression of all DR ligands, TNFR1, Fas and p75NTR but decreased levels DR3 and DR5. The levels of DR adaptor proteins like TRADD and TRAF2 (primarily regulate pro-survival pathways) are reduced. FADD, which primarily regulate cell death, its level remains unchanged. RIPK1, which regulate pro-survival, apoptosis and necroptosis, its expression and proteolysis (inhibits necroptosis but activates apoptosis) are increased.

**Conclusions:**

The findings from the present study provide evidence towards the involvement of DR3, DR5, DR6, TL1A, TRAIL, TRADD, TRAF2, FADD and RIPK1 for the first time in prion diseases. The knowledge obtained from this research discuss the possible impacts of these 16 differentially expressed DR factors on our understanding towards the multifaceted neuropathology of prion diseases and towards future explorations into potential targeted therapeutic interventions for prion disease specific neuropathology.

**Supplementary Information:**

The online version contains supplementary material available at 10.1186/s12967-024-05121-x.

## Background

Prion diseases are a group of irreversible and transmissible neurodegenerative diseases, which comprises scrapie in sheep and goat, bovine spongiform encephalopathy (BSE) in cattle, and various human disorders like Creutzfeldt-Jacob disease (CJD) [1–4]. Noted by the absence of nucleic acid, these diseases defy conventional pathogen paradigms by inducing the conformational transformation of normal cellular prion protein (PrP^C^) into a misfolded, infectious isoform (PrP^Sc^) [2, 3, 5]. Under physiological condition, this alteration leads to accumulation of PrP^Sc^ aggregates or amyloid plaques, driving spongiform degeneration and neuronal loss [4, 6, 7]. This was elegantly proved by grafting neural tissue overexpressing PrP^C^ into the brain of PrP^C^-deficient mice that are resistant to infection from prion. The results from this experiment have shown that, neuropathology and infectivity related to PrP^Sc^ are restricted to PrP^C^ expressing grafted tissue [8, 9]. Therefore, the expression of PrP^C^ is pivotal for disease progression and underscores the threshold-dependent neurotoxicity of PrP^Sc^.

The activation of microglial cells and astrocytes are other hallmark neuropathology of prion diseases [10]. Accumulation of PrP^Sc^ and activation of glial cells closely precede neuronal death in prion disease [4]. Normally, the activated microglia respond to neuronal damage and removes debris through phagocytosis. Chronic activation of these cells leads to the release of various cytotoxic molecules such as proinflammatory cytokines, reactive oxygen species, proteases and complementary proteins that are detrimental to healthy neurons in prion disease [10]. Nevertheless, the specific molecular and cellular pathways underlying the neuropathology of prion disease remain partially defined.

Three types of programmed cell deaths are discriminated: (1) apoptosis, (2) autophagy and (3) cytoplasmic cell death [11]. Reports on autophagy is limited and suggest more towards impairment [12–16] than activation [17, 18] of autophagy in prion disease. A single study suggests the cell death through necroptosis than apoptosis of scrapie-infected SMB-S15 cell line to LPS-activated microglia supernatant and TNFα (tumor necrosis factor-α) stimulations. This study undermines the importance of other cytokines present in the supernatant of activated microglia, which also contain LPS making it not a logical model of prion disease [19]. Therefore, conflicting data on autophagy and limited studies on cytoplasmic cell death leave critical gaps in our understanding, particularly regarding the dominant cell death pathway/s mediating the neuropathological conditions of prion diseases.

The misfolding of PrP^C^ is reported to produce neurotoxic stimuli, potentially through oligomerization of PrP^C^ on the cell surface and interacting with unknown transducing partners [20, 21]. Moreover, PrP^Sc^ aggresome has been implicated in the activation of caspase-8 and caspase-3, resulting in apoptosis. Collectively, the synthesis of proinflammatory cytokines by activated microglia, oligomerization of PrP^C/Sc^ on cell surface and activation of caspase-8 defines the involvement of death receptor pathways in the neuropathology of prion diseases. This complex and multifaceted nature of prion disease neuropathology recommends further studies, particularly regarding the role of cell death pathways and their regulation.

Six major death receptors (DRs) have been defined in human: TNF receptor 1 (TNFR1/DR1), FS-7-associated surface antigen (Fas/DR2/CD95), DR3, TNF-related apoptosis-inducing ligand (TRAIL) receptor 1 (TRAILR1/DR4), TRAILR2 (DR5) and DR6 [22, 23]. Among TRAILRs, rodents harbour only TRAILR2 in their genome [24]. These receptors are single-pass, type-1 transmembrane protein containing a conserved 80-amino acids sequence, referred as death domain (DD) [22, 25]. Additionally, the nerve growth factor (NGF) receptor (NGFR/p75^NTR^) and ectodysplasin a receptor (EDAR) also contains a DD, thereby categorizing them within the DR cohort [22, 26]. Unlike other DRs, EDAR primarily diverts in the activation of pro-inflammatory and pro-survival, pathways [26].

Each DR is specific to its cognate ligand: TNFR1 binds TNFα, Fas associates with FasL, DR3 engages with TL1A, TRAILR1 and TRAILR2 interact with TRAIL, and p75NTR binds either NGF or PrP^Sc^. However, the ligand for DR6 is not fully defined, hence remains uncertain [24]. The functions of DRs depend on their interaction with ligand and adaptor proteins. Receptors like TNFR1, DR3, DR6, and p75NTR, form complexes (known as complex I) with TRADD (TNFR1-associated death domain), RIPK1 (receptor-interacting protein kinase 1), and TRAF2 (TNFR-associated factor 2), orchestrating responses that range from inflammatory to pro-survival via NF-κB JNK and MEKK pathways. Failure to form complex I, results in the assembly of death-inducing stimulating complex (known as DISC or complex II), which involves the intracellular aggregation of either TRADD, RIPK1, FADD (Fas-associated death domain) and caspase-8/10 or RIPK1, FADD and caspase-8/10 leading to apoptotic cell death [23, 27]. Conversely, receptor such as Fas, and TRAILRs, upon ligand binding, initiate programmed cell deaths by forming DISC (known as complex I). DISC in this case is a complex of intracellular DD of DRs, FADD and caspase-8 or-10 [28]. While apoptosis is the major signalling outcome of Fas and TRAILR activation, they can also activate cell survival pathways by recruiting TRADD-RIPK1-TRAF2 or RIPK1-TRAF2 complexes (known as complex II) [29]. The nuanced, pleiotropic functions of DRs, underscored by their redundancy and complex signaling pathways, necessitate an in-depth study at polygenic level to fully elucidate their implications in the neuropathology of prion diseases.

The volume of research delineating the role of death receptors (DRs) in prion disease remains remarkably limited. Available reports often present an incomplete picture, failing to comprehensively address the intricacies of all primary DRs, their cognate ligands, adaptor proteins, and critical post-translational modifications such as glycosylation and ectodomain shedding. Glycosylation of DRs is known to enhance their functional maturation and interaction with specific ligand resulting in its activation. Conversely, ectodomain shedding of mature DRs are known to inhibit their activation. Notably, literature on TNFR1 in prion disease is limited to a mere three reports and with just a single study on its soluble form [30–32]. Despite the pivotal role of inflammation and TNFα in these diseases, inconsistencies challenge the findings, leaving significant gaps in understanding [33–38].

Like TNFR1, the literature on Fas and FasL is scant in prion diseases and with inconsistency [32, 39–42]. Additionally, while overexpression of p75NTR is documented in certain prion disease models, the data remain conflicting, adding to the complexity [43–45]. The situation is similar for its ligand, NGF, where mere three reports offer contradictory perspectives [44, 46, 47]. Research on other death DRs like DR3 and DR5, and their ligands such as TL1A and TRAIL respectively is even more barren, with no studies describing their roles in prion neuropathology. A similar landscape prevails for DR6.

Interestingly, not a single study so far has focused into the crucial role of TRADD, TRAF2, FADD, and RIPK1 in prion diseases. This research gap is notable, given the fact that the interaction of DRs with their ligand and adaptor proteins intricately regulate cellular fate, transitioning between survival and apoptosis. To address these lacunae, a comprehensive analysis was performed using a C57BL/6J mouse model of prion disease, which exhibits disease-related neuropathology similar to human prion disease. This study meticulously examined the expression of six DRs with death domains (TNFR1, DR3, DR6, DR5, Fas, and p75NTR), scrutinizing their glycosylation, proteolysis status, and their specific ligands (TNFα, TL1A, TRAIL, FasL, proNGF/NGF, and PrP^Sc^) and adaptors (TRADD, TRAF2, FADD, and RIPK1).

The insights obtained from this research elucidate the possible impact of these 16 differentially expressed DR factors on our understanding towards the multifaceted neuropathological characteristics of prion diseases. Moreover, findings from this research may help in finding ways to restore DR3 and DR5 pathways, RIPK1, TRADD and TRAF2 expression as future research and possible clinical implications. Notably, deep brain stimulation of cortico-striatal pathway may restore motor deficit in prion patients that could be another clinical implication.

## Methods

### Ethics statement

All experiments conducted on animals are in accordance with guidelines approved by the committee for the purpose of control and supervision of experiments on animals (CPCSEA) of National Brain Research Centre (NBRC), (Regd. No. 60/GO/ReBi-S/Re-L/01/CPCSEA). All animal procedures were reviewed and approved by Institutional Animal Ethics Committee (IAEC) of NBRC, (NBRC/IAEC/2019/159). In addition, usage of mouse prion was reviewed and approved by Institutional Biosafety Committee (IBSC) of NBRC, (NBRC/IBSC/2015/04 and NBRC/IBSC/2020/05).

### Mice

C57BL/6J mice were used in this study, which express *Prnp*^*a/a*^-allele, similar to the prion inoculum derived from CD1 mice. These mice are fully penetrant for prion disease and manifest neuropathology similar to human and other prion diseases making it a suitable animal model to study prion disease. These mice were obtained from NBRC (Regd. No. 60/GO/ReBi-S/ReL/01/ CPCSEA). This mouse line is maintained as an inbreed line by NBRC animal facility. During the study, mice were kept at 22 ± 2^O^C, 60 ± 5% humidity, 12:12 h (hr) of light/dark cycle and given ad libitum of food and water.

### Prion inoculum

The mouse scrapie prion used in this study is a 10% (wt/vol) brain homogenate from clinically ill CD-1 mice [48, 49] and was obtained as a kind gift from Prof. George A. Carlson, McLaughlin Research Institute, Great Falls, Montana, USA. This mouse prion isolate was derived initially from RML Chandler prion strain [50].

### Antibodies

The following antibodies were used for western blot: anti-prion antibody (#MAB5424; Millipore; 1:2000); anti-FADD antibody (#04-1113; Millipore; 1:500); anti-TRAF2 antibody (#ABC47; Millipore; 1:10000); anti-GFAP antibody (#N1506; DAKO; 1:10000); anti-TNFR1 antibody (#AF-425-PB; R&D; 1:500); anti-DR3 antibody (#MAB 943; R&D; 1:10000);anti-DR6 antibody (#AF144; R&D; 1:500); anti-TL1A antibody (#MAB7441; R&D; 1:500); anti-TRAIL antibody (#AF1121; R&D; 1:500); anti-p75^NTR^ (#8238S; Cell Signaling Technology (CST); 1:1000); anti-TRADD antibody (#3694S; CST; 1:500); anti-TNFα antibody (#11948S; CST; 1:1000); anti-RIPK1 antibody (#3693S; CST; 1:1000); anti-caspase-8 antibody (#4790S; CST; 1:1000); anti-caspase-3 antibody (#9611S; CST; 1:1000); anti-β-actin antibody (#4972S; CST; 1:5000); anti-DR5 antibody (#SAB3500427; Sigma-Aldrich Inc; 1:500); anti-Fas antibody (#AF5342; Sigma-Aldrich Inc; 1:8000); anti-FasL antibody (#Ab15285; Abcam; (1:5000); anti-Glyceraldehyde-3-phosphate dehydrogenase (GAPDH) antibody (#SC-32,233; Santa Cruz; 1:10000); anti-NGF antibody (#MA5-32067; Thermo; 1:2500); anti-Sortilin antibody (#MA5-31438; Thermo; 1:2500); anti-TrkA antibody (#PA5-94959; Thermo; 1:1250); anti-Neurofilament 160/200 antibody (N2912; Sigma-Aldrich Inc; 1:15000). The following antibody was used for immunofluorohistocemistry: anti-GFAP antibody (#N1506; DAKO; 1:3000). Secondary antibodies conjugated with horseradish peroxidase (HRP) were purchased from Pierce or CST and Alexa-594 fluorophore was purchased from Molecular Probes, Invitrogen, USA.

### Inoculation of mouse prion in C57BL/6J mice

Approximately, 21–30 days old, male C57BL/6J mice were used in the current study. By this age, mice express PrP^C^ protein, the substrate for prion replication. The skulls of mice at this age are soft for easy injection. This prevents rupture of skull and subsequent infection from inoculation. Mice were divided into two groups. Mice in both groups were anaesthetized with ketamine (100 mg/Kg body weight) and xylazine (10 mg/Kg body weight) prior to inoculation. Eight mice in prion group were inoculated intracerebrally with 20 µl of 1% mouse prion inoculum and six mice in control group were inoculated intracerebrally with 20 µl of 1X sterile phosphate buffer saline (PBS) using separate Hamilton syringes with 30-gauge needles [49]. All the procedures were performed inside a bio-safety level-2 cabinet. Control and prion-infected mice were kept separately throughout the experiment. Mice were inspected once every week for the first month, two times per week for the second month and 3–4 times per week thereafter for morphological and behavioural abnormalities.

### Harvest of brain

Brains were harvested immediately after cervical dislocation of terminally sick prion-diseased mice and corresponding control mice. Brains were dissected sagittally into left and right hemispheres. Both these brain hemispheres were flash-frozen on an aluminium foil spread over ethanol-dry ice bath. Frozen brain hemispheres were transferred into cryo-tubes individually and stored at 80^O^C. Right brain hemispheres were used for biochemical analysis whereas left-brain hemispheres were used for histological analysis. After each isolation, carcasses were wrapped along with blotting papers used in a bio-safety bag, sealed and kept in a designated −80^O^C deep freezer. Finally, these are disposed-off by incineration as approved by Institutional Bio-safety Committee.

### Preparation of brain lysates

Right brain hemispheres were quickly removed from −80^O^C and submerged into ice-chilled, 2.5 ml of lysis buffer (10 mM Tris pH 7.5, 150 mM NaCl, 0.5% Triton X-100, and 0.5% NaDOC). Brains were triturated successively through smaller-gauge needles (18-20-22-25-27 gauge). Each brain lysates were divided into two parts, First part was aliquoted, stored at -80^O^C and used for the detection of PrP^Sc^ and glycosylation of death receptors. The second half was added with protease inhibitors cocktail and DNase-I to a final concentration of 1X and 5U/ml respectively, and incubated for 15 min (mins) on ice. Lysates were then added with EDTA, SDS, NaF and NaVO3 to a final concentration of 2 mM, 0.1%, 5 mM and 2 mM respectively. All these steps were performed on ice. The lysates were aliquoted and stored at -80^O^C. Protein concentration in the lysates was determined by the bicinchoninic acid assay as recommended by the manufacturer (Pierce, USA).

### Western blot

#### Detection of PrP^Sc^ and PrP^C^ in brain lysates

To establish the prion disease in mice brains, presence of proteinase-K (PK) resistant PrP^Sc^ along with PrP^C^ were determined as reported earlier [49]. Briefly, 300 µg of total proteins from each brain lysate were treated with PK for 1 h (hr) at 37^O^C. Reaction was terminated by adding Pefabloc (Fluka, USA) followed by centrifugation at 18,000 X g for 30 min at room temperature (RT). The pellets were suspended in 1X sample loading buffer followed by denaturation for five mins at 97^O^C and electrophoresed in 15% Tris-Glycine-SDS-polyacrylamide gels (SDS-PAGE). Proteins were transferred onto nitrocellulose membranes and immunoblotted with a mouse anti-PrP antibody. Visualisation of bands was performed using supersignal west pico chemiluminescent substrate (Pierce, USA). Blots were stripped and reprobed with anti-GAPDH antibody to normalize protein loading and transfer.

#### Detection of proteins other than PrP

Equal amounts of total proteins (∼ 30–45 µg) from each sample were resolved in SDS-PAGE as mentioned above and transferred onto nitrocellulose or PVDF (polyvinylidene difluoride) membranes. After blocking, membranes were immunoblotted with appropriate primary antibodies overnight (O/N) at 4^O^C. Application of appropriate HRP-conjugated secondary antibodies, visualisation of immunoblots and normalization of protein loading and transfer were performed as described above. Time-lapse images of chemiluminescent protein bands were captured using BioRad chemidoc + XRS with ImageLab system. Densitometric analysis of the protein bands was performed on unsaturated images using ImageLab software (version 3.0) and fold-change between control and prion samples were calculated after normalizing with GAPDH and/or β-actin band intensity.

#### Detection of glycosylation of death receptors

To examine the N-glycosylation of DRs, brain lysates were treated with PNGase F following manufacture’s protocol (New England Biolabs, USA) and publications [51, 52] with minor modifications. Briefly, 30 µg of total proteins from each brain lysate was denatured by adding 10X glycoprotein denaturing buffer to a final concentration of 1X and incubating at RT for 10 min. Samples were then added with 10X glycol-buffer, 10% NP-40 to a final concentration of 1X and 1% respectively. Finally, 1000 units of PNGase F were added and incubated at 37^O^C for 3 h. Equal amount of PNGase F-untreated lysates along with corresponding deglycosylated lysates were resolved in 10% SDS-PAGE and immunoblotted as described above.

### Brain sectioning

Cryo-frozen left-brain hemispheres were transported from − 80^O^C to cryotome over dry ice and mounted onto a chuck. Frozen brain sections of 10 μm thickness were cut and transferred onto clean glass slides either uncoated or coated with (3-Aminopropyl)triethoxysilane (APTES). Sections on uncoated glass slides were quickly transferred to -80^O^C for storage. Sections on APTES-coated glass slides were immediately fixed in 4% paraformaldehyde in 1X PBS for 15 min at RT, followed by three washes in PBS for 5 min each. Sections were then dehydrated serially in 30%, 50%, 70%, 90% and 100% ethanol bath for 5 min each followed by another 100% ethanol bath for 5 min. Dehydrated sections were air dried and stored in sealed slide boxes for immunohistochemistry.

### Brain histoblots

Brain histoblots were prepared as described previously for cell blots [49]. Brain sections adherent to uncoated glass slides, stored at -80^O^C were quickly thawed and transferred onto a nitrocellulose membrane saturated with lysis buffer (10 mM Tris, pH 7.5; 150 mM NaCl; 0.5% sodium deoxycholate and 0.5% Triton X-100) for 1 min. The membrane was left on the blotting paper stack for another 5 min after removing the slides gently. Each membrane contained one control and one prion brain section and were allowed to dry completely for 1 h at RT and stored at -30^O^C for further experiment. To detect either PrP^Sc^ or PrP^C^, the blots were rehydrated in TBST, stained with ponceu-S and scanned to validate uniform tissue transfer. Blots were then destained completely in plenty of TBS and either left undigested to detect PrP^C^ or incubated with PK to detect PrP^Sc^. The PK digestion was terminated by rinsing the blots with TBST for 3 times, 5 min each, followed by incubating the blots for 30 min in TBST containing 3 mM phenylmethylsulfonyl fluoride (PMSF). Finally, the blots were incubated in 3 M GdnSCN (guanidine thiocyanate) in 10 mM Tris HCl, pH 7.8, for 10 min. Blots were rinsed 3 times in TBST for 10 min each followed by immunoblotting with mouse anti-PrP antibody and HRP-conjugated anti-mouse IgG antibodies. Blots were developed by NovaRed substrate (Vector Biolabs, USA). Blots were scanned and digital images were displayed with equal display setting for each pair of brain sections.

### Immunofluorohistochemistry

Three control and three prion brain sections of 10 μm thickness on APTES coated glass slides were used in this study. Protocol used was adopted from earlier publication on neurosphere sections [53]. Brain sections were rehydrated in 1X PBS and permiabilized in 0.3% Triton X-100 in PBS followed by blocking with TBS containing 0.1% BSA and 5% normal goat serum. Brain sections were then incubated with 5 µg/ml of rabbit anti-GFAP antibody for O/N at 4^O^C. Sections were washed in TBS containing 1% BSA and incubated with Alexa 594-conjugated goat anti-rabbit antibody for 1 h at RT. After washing 5 times, 5 min each, sections were fixed with 4% PFA for 15 min at RT. Sections were washed 3 times in PBS, 5 min each, rinsed once with water and mounted with anti-fade mounting medium containing DAPI.

### Image acquisition and analysis

Fluorescent images were captured using 40X lens and multi-dimension acquisition module in a Nikon Ti eclipse microscope supported by Metamorph software (version 7.7.0.0). Fluorescence images within an experiment were captured in one session with identical image acquisition settings and were displayed with equal image scale.

### Statistical analysis

The sample size of the present study is based on similarly published reports. Data shown represent three independent animals in each experimental group. The densitometry of desired band was measured from an unsaturated image of each factor and normalized with the intensity of GAPDH and/or β-actin. All histograms were presented as the means value ± standard deviations (SD) or errors of mean (SEM). The significance of difference between the mean values of the two groups was analysed by two-tailed, unpaired Student’s t-test using Microsoft EXCEL software and SigmaPlot software, version 12, Germany. One-way ANOVA was performed for multiple comparisons when data passed normality and equal variance test. When data failed the above tests, the groups were compared by non-parametric Kruskal-Wallis one-way ANOVA on ranks using SigmaPlot software. *P* ≤ 0.05 is considered statistically significant.

## Results

### Establishment of prion disease in C57BL/6J mice

**Fig. 1:**
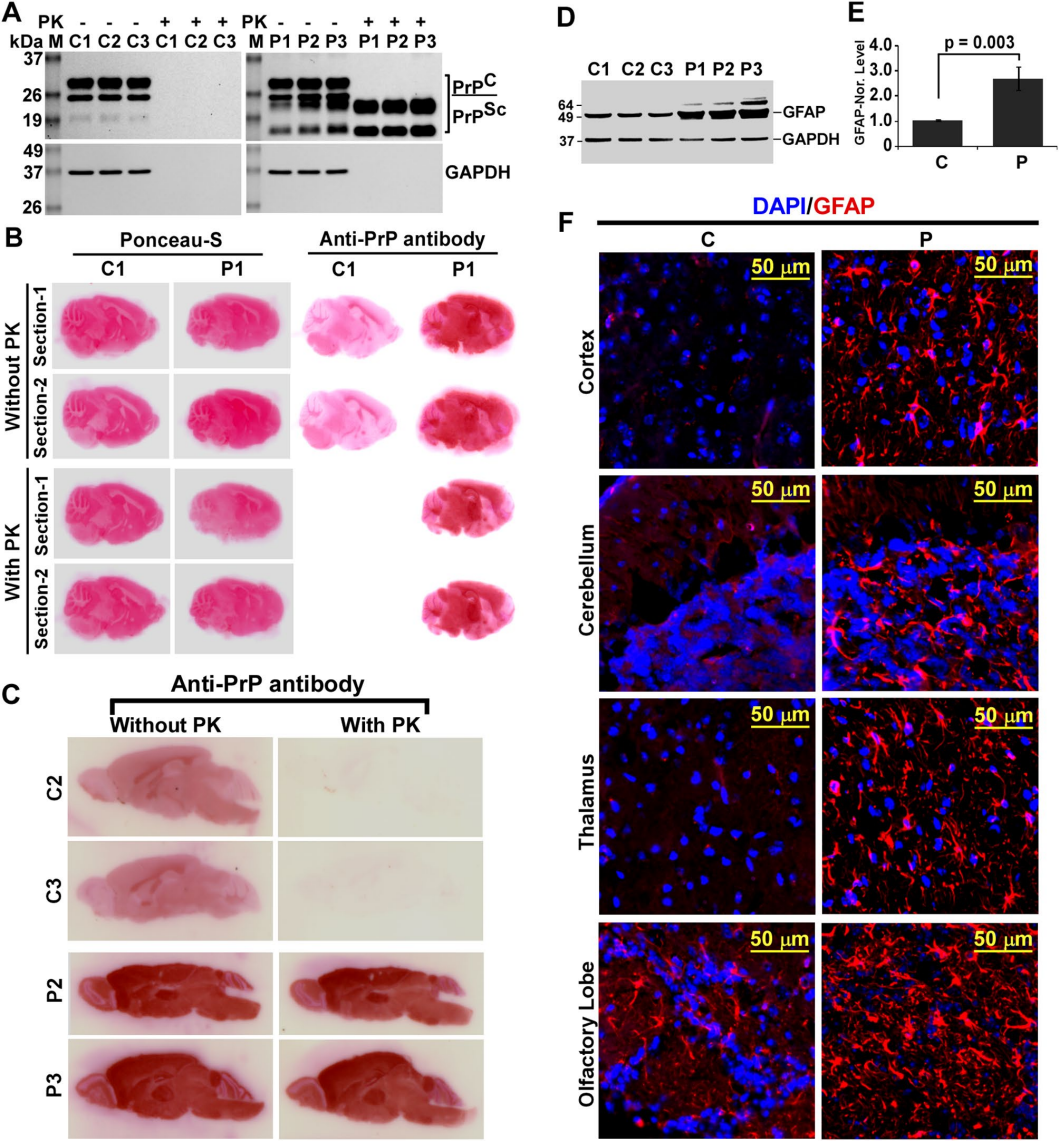
Characterization of neuropathologies in C57BL/6J mouse prion disease. **A** Western blot analysis of PrP^C^/PrP^Sc^ in control (C) and prion-infected (P) mice brain homogenates (MBH). Left panel indicates presence of PrP^C^ and GAPDH in normal MBHs, which were degraded upon proteinase-K (PK) treatment. In right panel, both PrP^C/Sc^ and GAPDH are observed in samples without PK treatment but only PrP^Sc^ but no GAPDH in PK-treated prion MBHs. **B, C** Histoblot analysis of three control and three prion infected mice brain sections shows increased PK-resistant PrP^Sc^ in all prion-infected but none in any control mice brain sections. **D, E** Prion-diseased mice brains show increased GFAP level than control mice. Each histogram represents mean ± standard deviation (SD) of a set of three mice brains from control and prion groups. *P* ≤ 0.05 is considered statistically significant (two-tailed unpaired *t*-test). **F** Immunofluorohistochemical analysis shows increased astrogliosis (GFAP + ve cells) in prion-infected mice brains than controls. DAPI: 4’,6-diamidino-2-phenylindole. Collectively prion infected mice exhibit robust neuropathological features of prion disease

All eight prion-infected mice survived the inoculation process while one mouse from control group died during inoculation. Prion-infected mice started showing morphological alterations such as hunch back posture, plastic tail (Additional file 1: Fig. S1) and body weight loss by 4 months post infection while control mice remained healthy. Additionally, infected mice exhibited defects in gait such as, frequent circling (Additional file 2: Video. S1, S2), repeated jumping at the edge of cage (Additional file 2: Video. S3, S4), defects in hind leg movement (Additional file 2: Video. S5) and weakened forearm grip (Additional file 2: Video. S6, 7). Around 140 days, all infected mice exhibited shivering, twitching, withdrawal from food and unwilling to move than corresponding controls (data not shown). Western blot analysis of control mice brain lysates without PK treatment shows PrP^C^ expression, which degraded completely by PK digestion (Fig. 1A, left panel). Prion-infected mouse brain lysates without PK treatment show increased level of PrP^C/Sc^ and prominent PrP^Sc^ bands post PK-treatment than control lysates, suggesting PK-resistant PrP^Sc^ accumulation restricted to diseased mice brains (Fig. 1A, right panel). Compared to controls, prion-diseased mice brain histoblots exhibit PK-resistant PrP^Sc^ accumulation all over the brain but prominently in thalamic, hippocampal, cortical, cerebellar and midbrain regions (Fig. 1B, C). Overexpression of GFAP is an established marker of astrocytosis. Western blot analysis exhibits 2.75 times higher and significant (*p* = 0.003) GFAP expression in prion-diseased mice brains than controls (Fig. 1D, E). Furthermore, increased GFAP positive cells with higher GFAP intensity are observed in most parts of the diseased brain than healthy controls (Fig. 1F). Moreover, western blot analysis of neurofilament-H (an established marker of mature neuron) shows significant reduced expression in prion-diseased brains than controls (data not shown). This loss of neurofilament-H expression might be due to neuronal loss, hence represents neurodegeneration seen in prion diseases. Collectively these data illustrate robust neuropathological features of prion disease in C57BL/6J mice and paving the way to study death receptor (DR) biology in prion disease.

### Involvement of extrinsic caspase cascade in C57BL/6J mouse prion disease

**Fig. 2:**
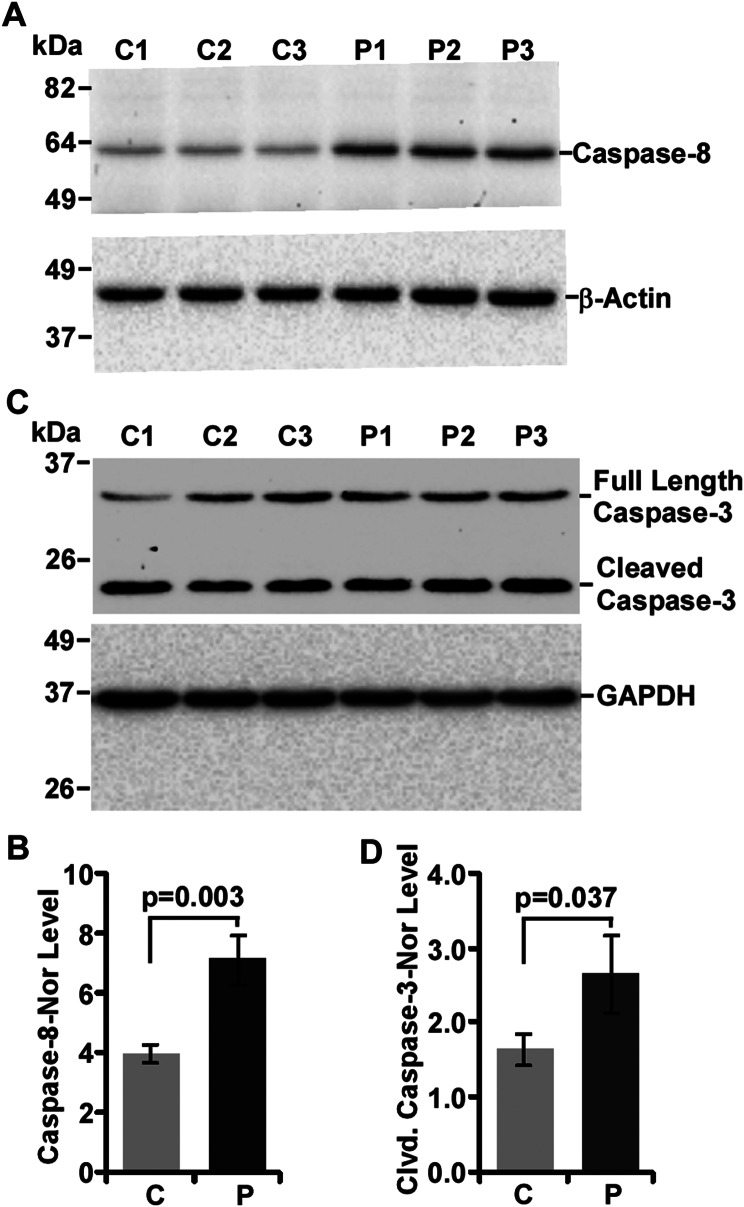
Altered expression of caspase-8 and caspase-3 in control (C) and prion-diseased (P) mice brains. **A, B** Prion-diseased mice brain lysates have increased caspase-8 level than controls. **C, D** Prion-diseased mice brain lysates have increased cleaved caspase-3 level than controls. GAPDH and β-actin were used as loading controls. Each histogram represents mean ± SD of a set of three mice brains from control and prion groups. *P* ≤ 0.05 is considered statistically significant (two-tailed unpaired *t*-test). Taken together, both caspase-8 and caspase-3 are activated in prion disease

Activation of extrinsic caspases such as caspase-8 and/or -10 are indicators of death receptors activation. Since caspase-10 gene is absent in mouse [54], only caspase-8 was examined in this study. Western blot image of caspase-8 demonstrates overexpression of caspase-8 in all prion-diseased mice brains than controls (Fig. 2A). Densitometry of caspase-8 shows 1.8 times and significant (*p* = 0.003) higher expression of caspase-8 in prion mouse brain lysates than controls (Fig. 2B). Downstream target of caspase-8 is caspase-3. Western blot image of caspase-3 shows increased proteolysis of caspase-3 (∼ 20-kDa) in prion-diseased brains than controls (Fig. 2C). Cleaved caspase-3 intensity is 1.6-times increased in prion-diseased brain lysates than controls and statistically significant (*p* = 0.037) (Fig. 2D). Collectively, activation of both caspase-8 and caspase-3 suggest the involvement of death receptor pathways in prion disease.

### Alteration of TNFR1 expression in mouse prion disease

**Fig. 3:**
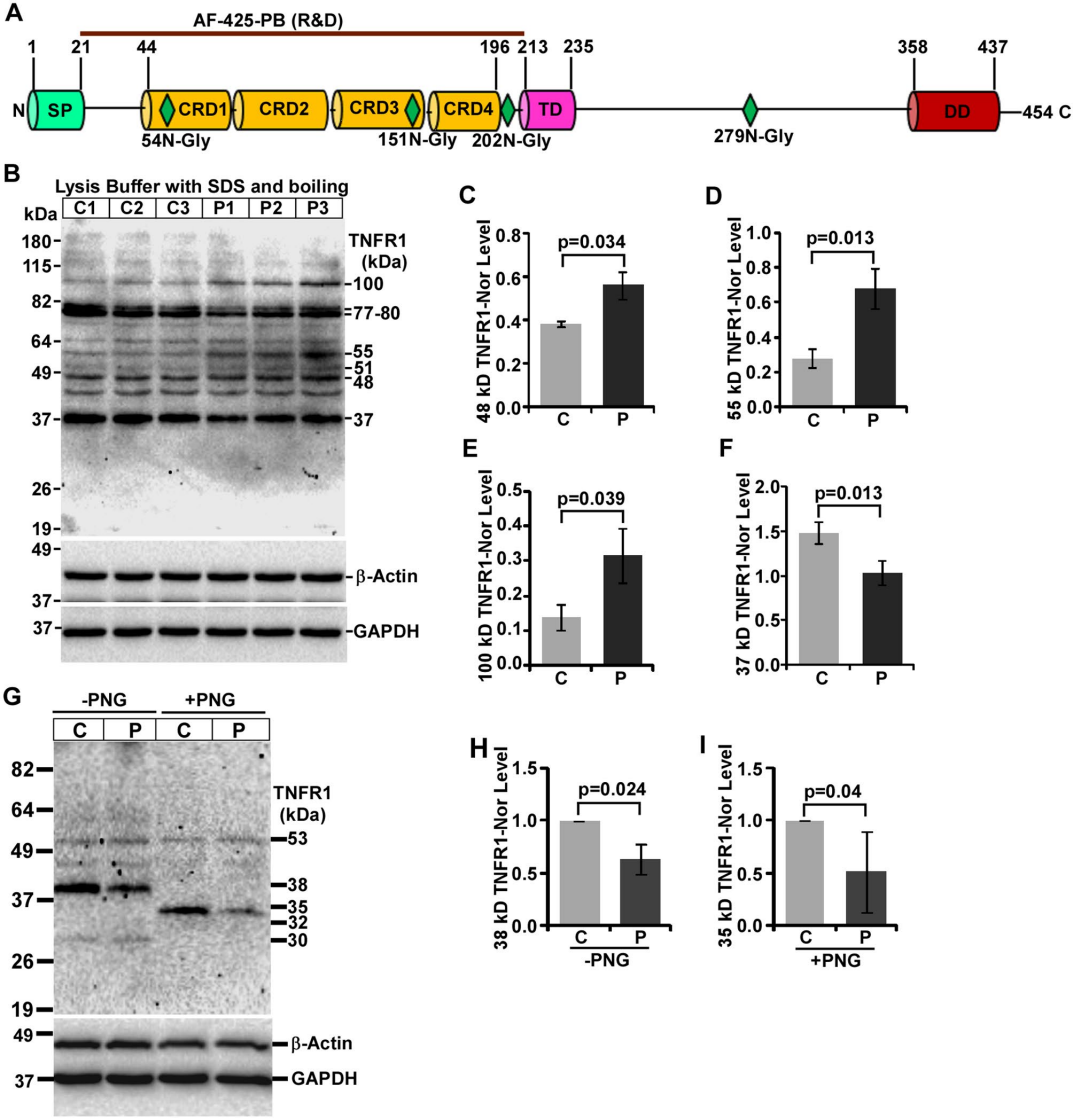
Expression of TNFR1 isoforms are altered in C57BL/6J mouse prion disease. **A** Schematic structure of TNFR1 protein shows signal peptide (SP), cysteine-rich domains (CRD), transmembrane (TM), death domains (DD), glycosylation sites and epitope of anti-TNFR1 antibody. **B** Western blot analysis of TNFR1 on lysates from control and prion-diseased mice brains. **C** The densitometry of 48-kDa, **D** 55-kDa and **E** 100-kDa TNFR1 isoforms show significant increased expression in Prion-diseased (P) mice brains than controls (C). **F** Contrarily, 37-kDa TNFR1 band intensities are significantly decreased in same setting. **G** Western blot analysis of TNFR1 on lysates deglycosylated by PNGase F (PNG) show 38 and 45 kDa TNFR1 isoforms are shifted to 35 kDa. **H** The densitometry of 38-kDa, **I** 35 kDa TNFR1 bands show reduced expression in prion-diseased mice brains than controls. GAPDH and β-actin were used as loading controls. Each histogram represents mean ± SD of a set of three brain lysates from control or prion-diseased mice. Two-tailed unpaired *t*-test was performed to compare the significance of difference between two groups (for detail, see materials and method section). *P* ≤ 0.05 is considered statistically significant

TNFR1 is the most widely studied DR protein that undergoes ectodomain shedding, glycosylation and oligomerization suggesting coexistence of its multiple isoforms, which are hardly studied in prion diseases. In order to detect all these isoforms of TNFR1 protein, application of western blot analysis is logically preferred over ELISA and Q-RT-PCR. Therefore, to study TNFR1 protein, western blot analysis was employed in this study. Antibody used to detect TNFR1 was developed against 22–212 amino acid long fragment of TNFR1 which can detect both full-length (48-55-kDa), soluble (33-kDa), and their oligomeric and glycosylated TNFR1 isoforms (Fig. 3A) suggesting the display of multiple TNFR1 bands in western blot. The results from western blot analysis on denatured brain lysates show multiple TNFR1 bands (isoforms) ranging from 37-100-kDa. Image shows higher intensity of full-length (FL)-TNFR1 bands (48-55-kDa) in all prion-diseased mice brains than controls and 55-kDa band as the prominent one (Fig. 3B). The densitometry shows significant (*p* < 0.05) increased levels of 48-, and 55-kDa TNFR1 isoforms by 1.45, and 2.45 times respectively in diseased brains than controls (Fig. 3C, D). Similarly, the level of 100-kDa band is 2.287 times higher and significant (*p* = 0.039) (Fig. 3B, E), whereas the level of 37-kDa band (Fig. 3B, F) is significantly (*p* = 0.013) decreased by approximately 31% in prion-diseased brain lysates than controls. Additionally, 77-80-kDa bands, which are reactive to TNFR1 antibody, are decreased by 27% in diseased mice brains compared to controls (Fig. 3B) but without statistical significance (graph not shown). To address the glycosylation status of TNFR1, native brain lysates (without SDS) were treated with PNGase-F (for protocol see method). The results clearly exhibit the shift of 38-kDa TNFR1 to 32-35-kDa, whose intensities are also reduced in diseased mice brains than controls (Fig. 3G). The densitometry of 38- and 35-kDa bands exhibits significant (*p* < 0.05) reduction by 37% and 49% respectively (Fig. 3H, I) suggesting TNFR1 is glycosylated and cleaved to form 38-kDa soluble TNFR1. Although the use of western blot analysis is useful to identify and quantify various isoforms of protein, like TNFR1 and other DRs but can be limited in understanding direct association of these factors with specific neuropathology. Nevertheless, the data show increased FL-TNFR1 isoforms but decreased neuroprotective soluble TNFR1 isoforms.

### Alteration of Fas expression in mouse prion disease

**Fig. 4:**
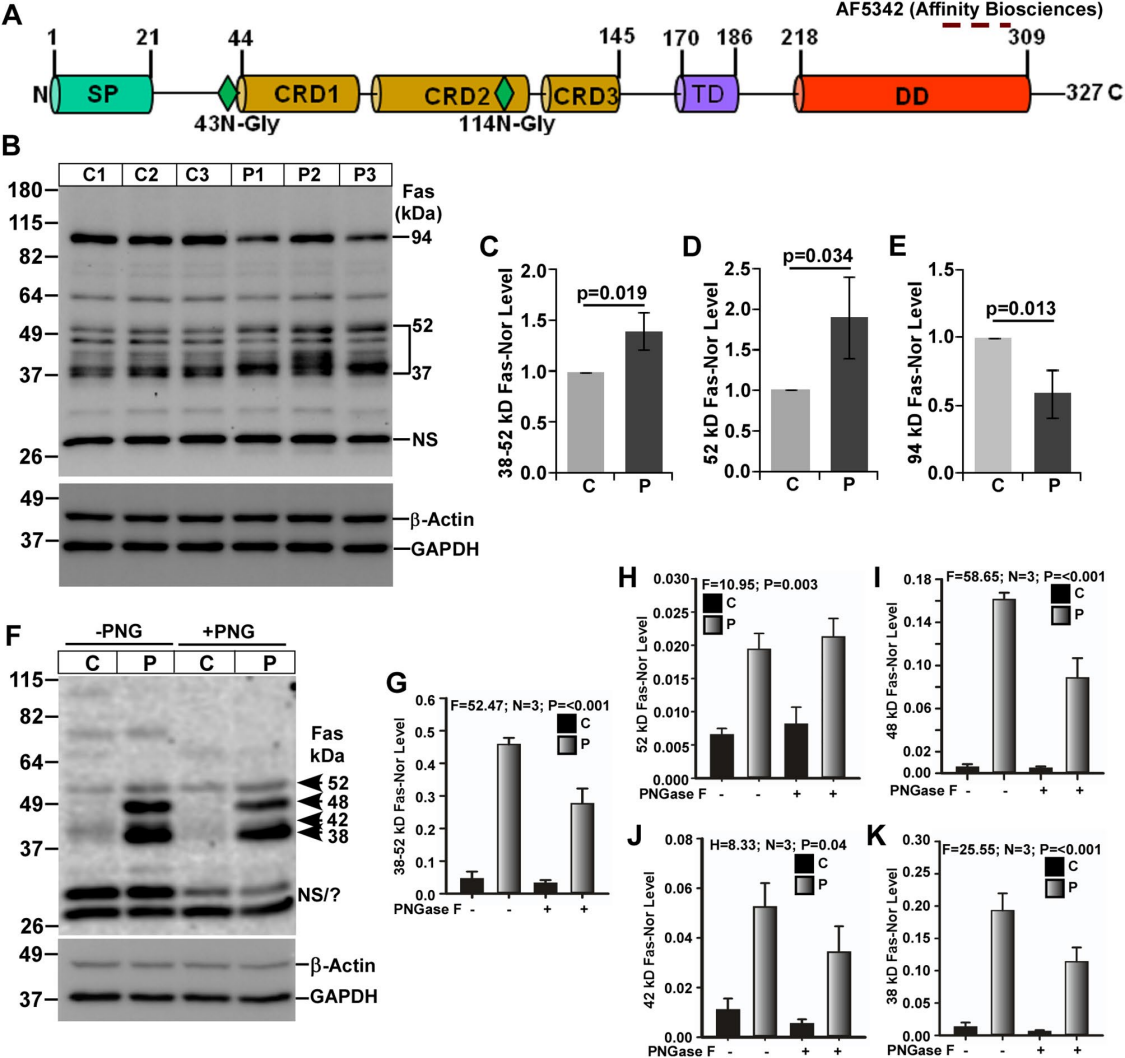
Expression of Fas isoforms are altered in C57BL/6J mouse prion disease. **A** Schema of Fas protein structure shows various domains as mentioned for TNFR1 protein. Glycosylation sites and epitope of anti-Fas antibody are also shown. **B** Western blot analysis of Fas protein on lysates from control (C) and prion-diseased (P) mice brains. **C** The densitometry of 38-52-kDa and **D** 52-kDa Fas isoforms show significant increased expression in prion-diseased mice brains than controls, whereas, **E** 94-kDa Fas isoform shows significant reduction in same setting. **F** Western blot analysis of Fas on lysates deglycosylated by PNGase F (PNG) show increased levels of 38–52 kDa but decreased level of 94 kDa Fas isoform in prion-diseased mice brains than controls. **G** The densitometry of 38- 52-kDa together, **H** 52-kDa, **I** 48-kDa, **J** 42-kDa and **K** 38-kDa Fas isoforms exhibit reduced intensity for 38-, 42- and 48- but not for the 52-kDa Fas isoforms in PNGase F treated sample than untreated samples. Intensities of these bands from PNG-treated or -untreated are higher in prion group than control group. GAPDH and β-actin were used as loading controls. Each histogram represents mean ± SD (C-E) or SEM (G-K) of a set of three brain lysates from control or prion-diseased mice. Two-tailed unpaired *t*-test was performed to compare the significance of difference between two groups and one-way ANOVA for multiple groups as described in materials and method section. *P* ≤ 0.05 is considered statistically significant

Mature or full-length (FL)-Fas is a 35-kDa DR protein derived from a 37.4-kDa nascent Fas protein, which has one signal peptide (SP), three cysteine-rich domain (CRD), one transmembrane (TM), one death domain (DD) and two N-glycosylation sites. To study Fas expression in the present mouse prion disease, an anti-Fas antibody specific to its C-terminal region was used in western blot analysis. This antibody can detect glycosylated and oligomeric FL-Fas (35-70-kDa) and C-terminal fragment (∼ 20-kDa) and its oligomeric form of Fas (Fig. 4A). The logical explanation for application of western blot analysis is similar to TNFR1 protein and other DRs. Results from western blot analysis using denatured brain lysates demonstrate multiple bands around 37-52-kDa, which are increased in all prion-diseased mice brains than controls (Fig. 4B). Among them, the most prominent one is 52-kDa Fas isoforms. Densitometry of combined intensity of 37-52-kDa Fas bands and 52-kDa Fas isoform show a significant (*p* < 0.05) increased level by ∼ 39% (Fig. 4C) and 90% (Fig. 4 D) respectively in prion lysates than controls. Such high molecular weight Fas isoforms could be due to its glycosylation. Conversely, the intensity of 94-kDa Fas isoforms decreased significantly (*p* = 0.013) by 42% (Fig. 4B, E) in diseased brain lysates than controls. The result from the deglycosylation experiment by PNGase F treatment clearly shows four prominent bands around 38-52-kDa and two bands around 28-32-kDa. The 38-52-kDa bands demonstrate a clear increased intensity in all diseased brains than controls (Fig. 4F). Densitometry of combined intensity of 38-52-kDa bands shows a significant (*p* = 0.015) increased level by ∼ 5.8-fold in prion-diseased brain lysates than controls (Fig. 4G). Interestingly, the intensity of 52-kDa bands is not decreased upon PNGase F treatment and its densitometry demonstrates a marginal 6% decreased level with *p* = 0.77 between PNGase F-untreated and treated groups, suggesting 52-kDa Fas isoforms is not N-glycosylated. Moreover, 52-kDa Fas isoforms level is significantly higher in all prion-diseased brains than controls (Fig. 4H). Intensity of 38-48-kDa bands are decreased visibly in PNGase F-treated samples than untreated samples suggesting these Fas isoforms are N-glycosylated (Fig. 4F). Densitometry of 48-, 42- and 38-kDa bands intensity decreased prominently but not significantly in PNGase F-treated prion brains lysates than controls. Similar to denatured samples, all these bands displayed significant increased level in prion-diseased mice brains than controls (Fig. 4I-K respectively). The 94-kDa band, which is clearly visible in denatured brain lysates is barely detected in these experiments. Furthermore, the intensity of 28- and 32-kDa bands are similar between control and diseased brain lysates, hence considered as nonspecific, or degraded C-terminal fragments of Fas. Taken together, the data show increased levels of Fas isoforms in the present prion mouse model.

### Alteration of DR3 expression in mouse prion disease

**Fig. 5:**
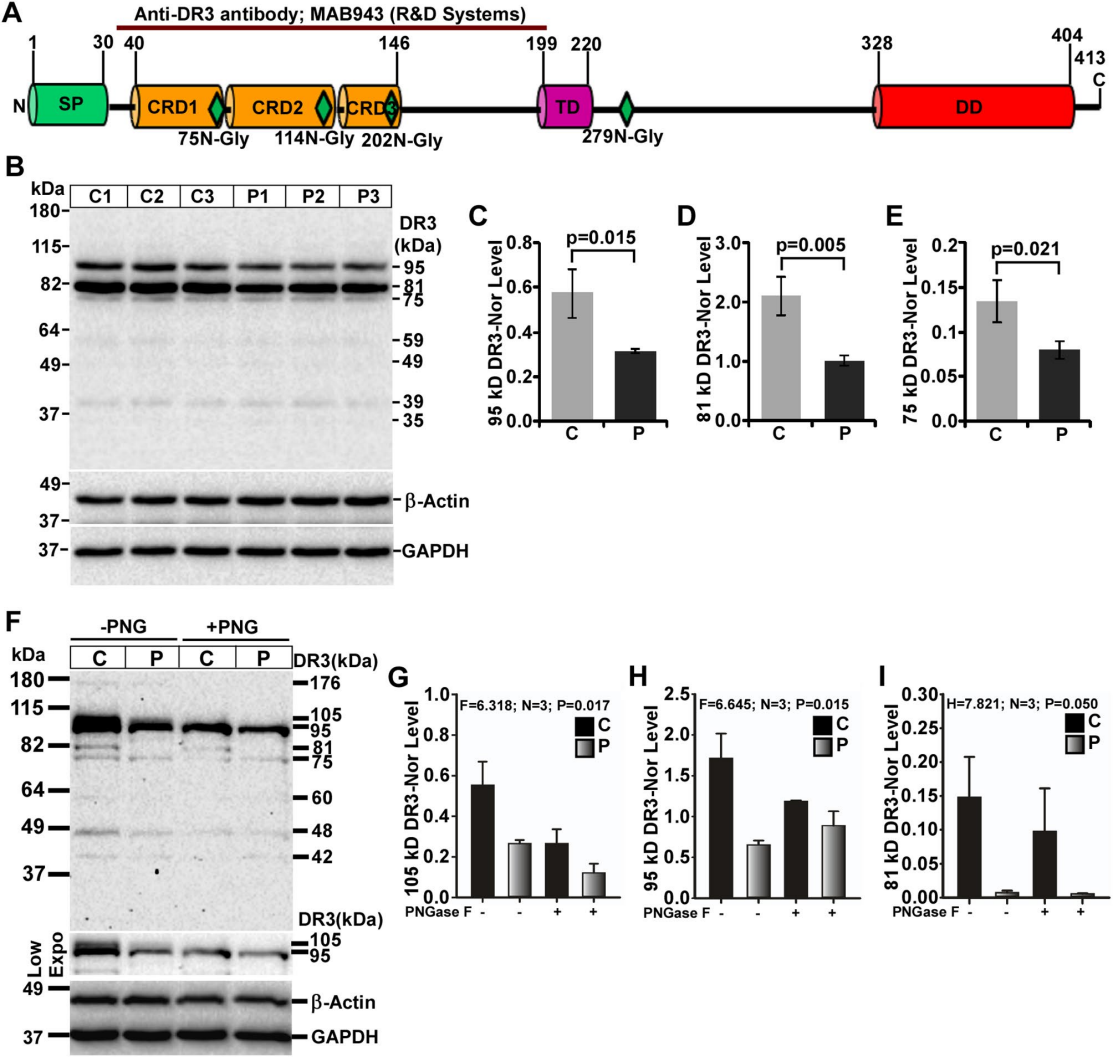
Decreased expression of DR3 protein isoforms in C57BL/6J mouse prion disease. **A** Graphical sketch of a 38.8 kDa DR3 protein structure showing various domains (as described earlier), glycosylation sites and epitope of anti-DR3 antibody. **B** Western blot analysis of DR3 on lysates from control (C) and prion-diseased (P) mice brains showing decreased expression pattern in all prion-diseased mice brains than controls. **C** The densitometry of 95-kDa, **D** 81-kDa and **E** 75-DR3 isoforms show significant reduced expression in prion-diseased mice brains than controls. **F** Western blot analysis of DR3 on PNGase F (PNG) treated lysates show reduced intensity of 176-, 105-, 81- and 48- kDa but not of 95-, 75- and 60-kDa DR3 isoforms than untreated lysates. **G** The densitometry of 105-kDa, **H** 95-kDa and **I** 81-kDa DR3 isoforms exhibit reduced intensity for 105- and 81- but not for the 95-kDa DR3 isoforms to PNGase F treatment. Intensities of these bands from PNG-treated or -untreated are remarkably less in prion group than control. GAPDH and β-actin were used as loading controls. Each histogram represents mean ± SD (C-E) or SEM (G-I) of a set of three brain lysates from control or prion-diseased mice. Two-tailed unpaired *t*-test was performed to compare the significance of difference between two groups and one-way ANOVA for multiple groups. *P* ≤ 0.05 is considered statistically significant

Mature DR3 is a 39-kDa, type-I transmembrane protein derived from a newly synthesized 41-kDa DR3 protein. DR3 has one SP, three CRDs, one TM, and one DD. The antibody used in this study is against extracellular domain of DR3 that can detect both full-length and soluble DR3 fragment (Fig. 5A). Western blot analysis on denatured brain lysates exhibits seven bands (95-, 81-, 75-, 60-, and 49-, 39- and 35-kDa). The intensities of all these bands show reduced levels, 95-, 81- and 75-kDa isoforms as the prominent ones in prion mice brain lysates than controls (Fig. 5B). The densitometry of 95-, 81- and 75-kDa DR3 bands shows significant (*p* < 0.05) reduced levels (Fig. 5C-E respectively) whereas 59-, 49- and 39-kDa DR3 bands also show non-significant reduced level in diseased over control brain lysates. DR3 is known to have four N-glycosylation sites (Fig. 5A). Western blot analysis of brain lysates with or without PNGase F treatment exhibits high molecular weight isoforms of DR3 ranging from 176-75-kDa. Intensity of these DR3 isoforms is reduced again in all prion brain lysates than controls (Fig. 5F). Intensity of 176-kDa DR3 band is barely detected but reduced in prion mice brains than controls. This band is not visible in PNGase F-treated samples, hence, excluded from densitometric evaluation. Contrarily, the intensity of 105-kDa DR3 band is significantly reduced in prion-diseased mice brains than controls and almost 50% reduced in PNGase F-treated samples (Fig. 5F, G), suggesting possible N-glycosylation of these DR3 isoforms. However, other high molecular weight DR3 bands (95- and 81-kDa) exhibit significant reduced level among prion mice brains than controls but without significant change between PNGase F-treated and untreated groups (Fig. 5F, H-I respectively) suggesting these DR3 isoforms are not N-glycosylated. The 81-kDa band which is prominently seen in denatured lysates are barely visible in native lysates, suggesting SDS favors the extraction of this DR3 isoforms. Moreover, 81-kDa DR3 band is significantly higher in control mice brain lysates than diseased brain lysates. (Fig. 5F, I). Collectively, the data obtained from both these experiments show the expression of DR3 isoforms are reduced in mouse prion disease.

### Alteration of DR5 expression in mouse prion disease

**Fig. 6:**
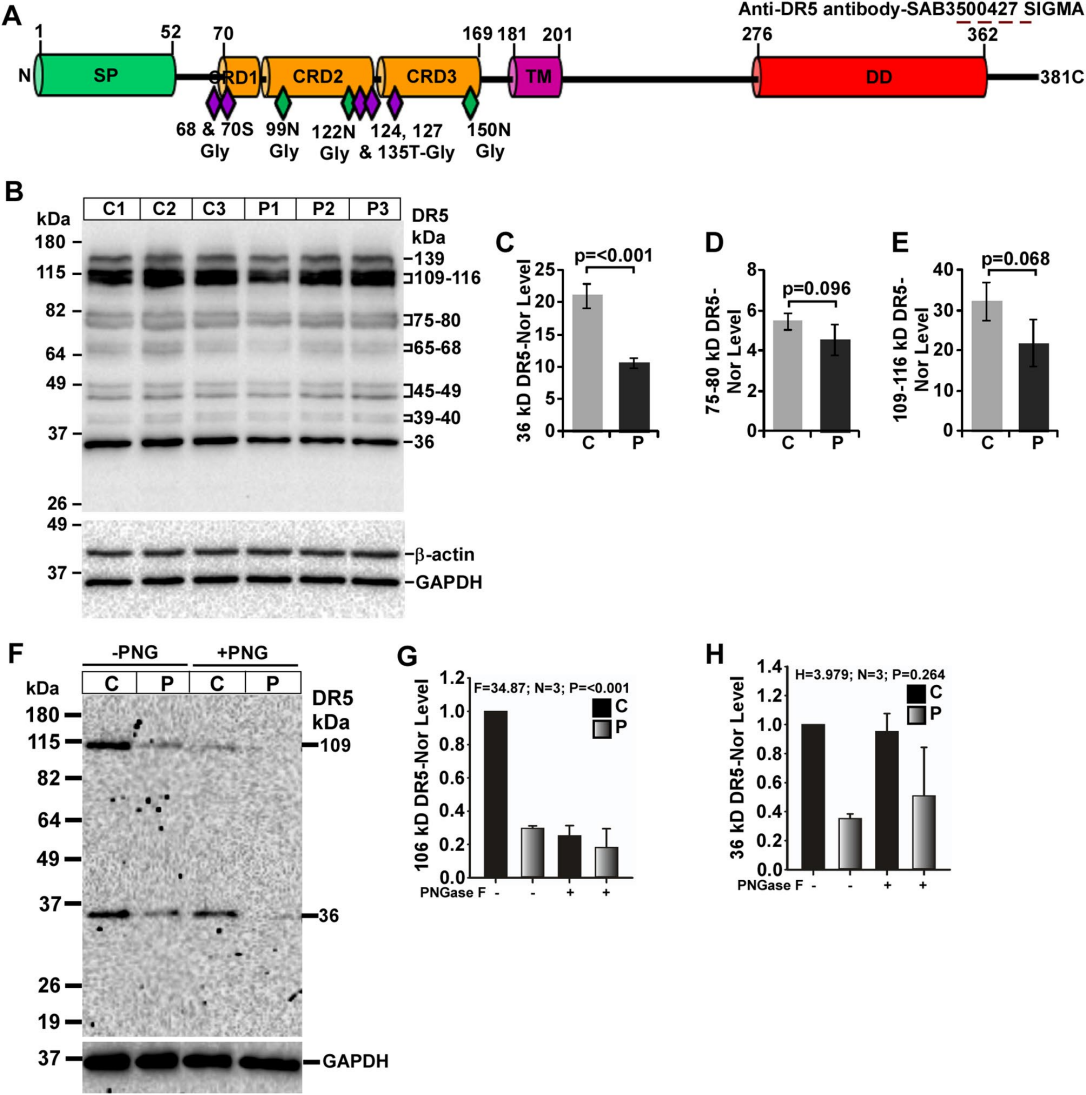
Expression of DR5 protein isoforms are reduced in prion-diseased (P) C57BL/6J mice brains than control (C) mice. **A** Schematic structure of a 36.6 kDa DR5 showing various domains, glycosylation sites and epitope of anti-DR5 antibody. **B** Western blot analysis of DR5 on lysates from control and prion-diseased mice brains showing decreased expression pattern of all DR5 isoforms with prominence for 36-and 109-116-kDa isoforms in all prion-diseased mice brains than controls. **C** The densitometry of 36-kDa, **D** 75–80 kDa and **E** 109–116 kDa DR5 isoforms show significant reduced expression for 36-kDa and reduced level for both 75–80 kDa and 109–116 kDa DR5 isoforms in prion-diseased mice brains than controls. **F** PNGase F (PNG)-treated brain lysates shows two bands, of which, 109-kDa DR5 isoform is sensitive to PNGase F but not the 36-kDa band. **G** The densitometry of 109-kDa band show significant reduced intensity upon PNGase F treatment. **H.** The densitometry of 36-kDa DR5 band show resistant towards PNGase treatment. Both these DR5 bands show reduced level in prion-diseased mice than controls. GAPDH and β-actin were used as loading controls. Each histogram represents mean ± SD (C-E) or SEM (G-H) of a set of three brain lysates from control or prion-diseased mice. Two-tailed unpaired *t*-test was performed to compare the significance of difference between two groups and one-way ANOVA for multiple groups. *P* ≤ 0.05 is considered statistically significant

DR5 is a 36-kDa mature DR protein derived from its nascent 42-kDa protein. It has one SP, three CRDs, one TM and one DD. DR5 is known to have multiple N- and O-glycosylation sites suggesting multiple DR5 bands from western blot analysis. To study DR5 in mouse prion disease, a C-terminal region specific anti-DR5 antibody was used which can detect both full-length and C-terminal but not the secreted/soluble DR5 fragments (Fig. 6A). Western blot analysis of DR5 expression in denatured brain lysates shows all DR5 bands are less intense in prion-diseased mice brains than controls, with 36- and 109-116-kDa bands as the prominent ones (Fig. 6B). The densitometry analysis demonstrates significant decreased level of 36-kDa mature DR5 band by 50% in diseased mice brains than controls (Fig. 6C). Other DR5 bands of 39-40-, 45-49-, 65-68- and 75-78-kDa show decreased level but without significance (Fig. 6D) in diseased mice brains than controls. The densitometry of 109-106-kDa bands exhibits decreased level (*p* = 0.068) in all prion-diseased mice brains by 33% than controls (Fig. 6E). The bands higher than 36-kDa, are possible glycosylated DR5 isoforms. Therefore, native brain lysates were subjected to PNGase F treatment followed by immunoblotting. The results exhibit two (36- and 109-kDa) visible bands. Intensity of both these bands is reduced in prion-diseased brains than controls (Fig. 6F). Between these two bands, 109-kDa band intensity is visibly decreased to PNGase F treatment. Densitometry of this band shows significant reduced intensity by 70% in PNGase F-treated than untreated control mice brain lysates (Fig. 6F, G), suggesting 109-kDa DR5 isoforms is mostly N-glycosylated. This DR5 isoform is also significantly decreased in prion-diseased mice brains than controls (Fig. 6G). In addition, similar to denatured lysates, native samples also exhibit significant reduction in 36-kDa band intensity by 65% in prion-diseased mice brain lysates than controls but its intensity remain unchanged to PNGase F treatment (Fig. 6H) suggesting 36 kDa DR5 isoform is not N-glycosylated. Collectively, the data obtained from this study clearly suggests the decreased expression of DR5 protein in mouse prion disease.

### Altered expression of DR6 in mouse prion disease

**Fig. 7:**
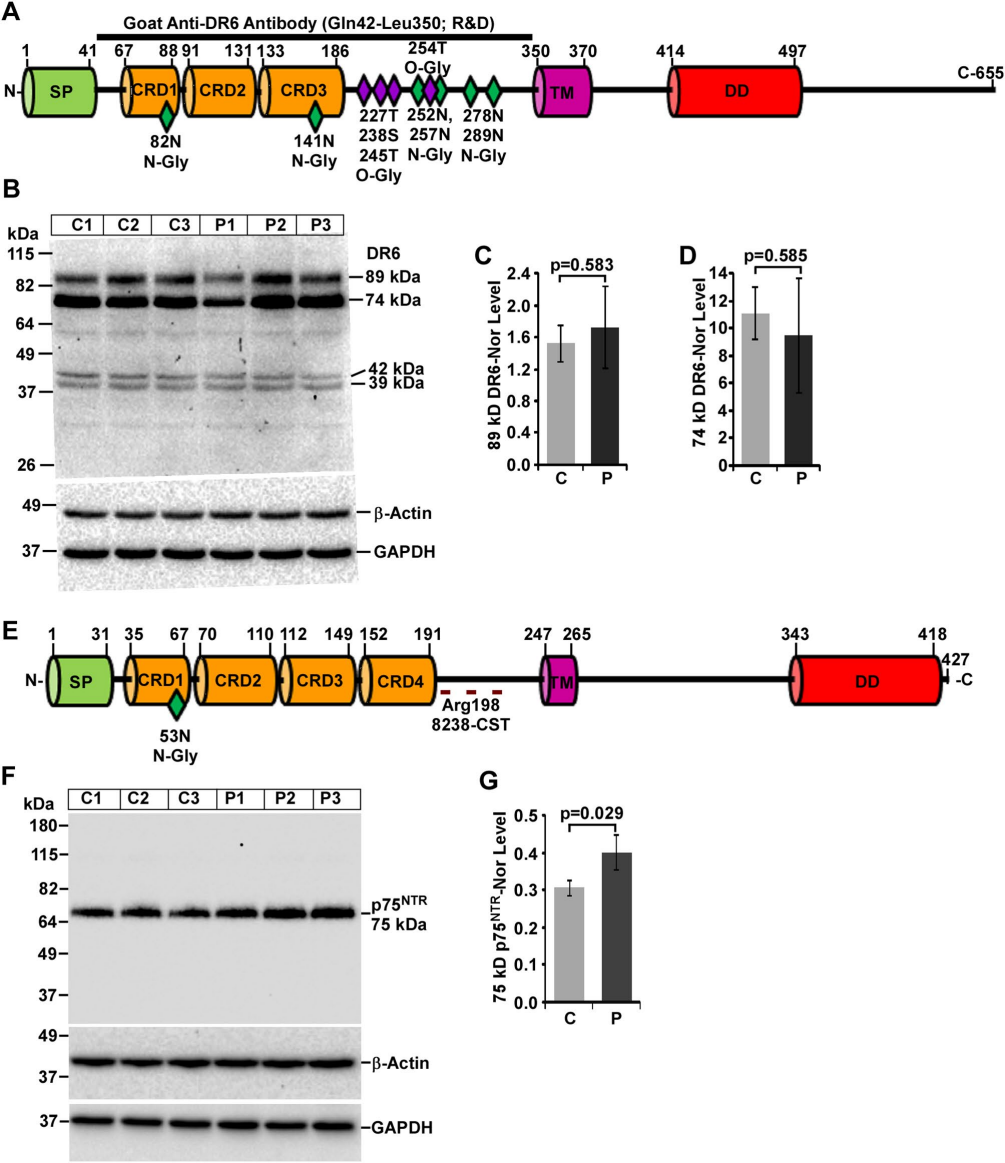
Expression pattern of DR6 and p75^NTR^ protein isoforms varies between control (C) and prion-diseased (P) C57BL/6J mice brains. **A** Schema of a 68-kDa DR6 protein structure shows various domains, glycosylation sites and epitope of anti-DR6 antibody. **B** Western blot analysis of DR6 on lysates showing similar expression pattern of all DR5 isoforms between control and prion-diseased mice brains. **D** The densitometry of 89-kDa, **D** 74-kDa DR6 isoforms show similar levels of expression within prion-diseased and control mice brains. **E** Schematic structure of p75^NTR^ protein exhibiting various domains, glycosylation sites and epitope of anti-p75^NTR^ antibody. **E** Western blot analysis of p75^NTR^ shows a dominant 75 kDa band with increased p75^NTR^ level in prion-diseased mice brains than controls. **F** The densitometry of 75 kDa band show significant increased intensity in prion diseased mice brain lysates than controls. GAPDH and β-actin were used as loading controls. Each histogram represents mean ± SD of a set of three brain lysates from control or prion-diseased mice. Two-tailed unpaired *t*-test was performed to compare the significance of difference between two groups. *P* ≤ 0.05 is considered statistically significant

Matured DR6 is a 68-kDa DR protein derived from its 72-kDa precursor having one SP, three CRDs, one TM, and one DD. Like other DRs, DR6 undergoes glycosylation at several places. The antibody used in this study was developed against the N-terminal of DR6 protein, which can detect both full-length and soluble DR6 with different glycosylation levels (Fig. 7A). Results show two prominent DR6 bands (89- and 74-kDa) and two faint bands (42- and 39-kDa) from denatured brain lysates. Unlike other DRs, difference in DR6 expression is not observed between prion-diseased mice brains and healthy controls (Fig. 7B). Densitometry of 89- and 74-kDa DR6 isoforms also exhibits no change between prion and control groups (Fig. 7C, D respectively). Since matured DR6 protein is of 68-kDa, 89- and 74-kDa bands are its possible glycosylated forms. Results from deglycosylation experiments of DR6 show, 94-kDa band is shifted to 80-kDa upon PNGase F treatment (Additional file 1: Fig. S2, A, upper panel) suggesting 94-kDa band is N-glycosylated DR6 isoform. Densitometry of these bands shows significant decrease in 94-kDa but not in 80-kDa (*p* = 0.47) (Additional file 1: Fig. S2, B, C) in prion mice brains than controls. Furthermore, additional bands at 50-, 46- and 42-kDa demonstrate no significant difference in expression level and remain unaffected to PNGase F treatment within prion and control groups. Taken together, these data suggest DR6 is highly N-glycosylated and its expression is not altered in mouse prion disease.

### Alteration of p75^NTR^ expression in mouse prion disease

A schematic representation of p75^NTR^ structure demonstrated SP, CRDs, TM, DD, and one N-glycosylation site. The antibody used in this study is around Arg-198 of human p75^NTR^, which can detect both full-length and proteolytic ectodomain of mouse p75^NTR^ (Fig. 7E). Western blot analysis of denatured brain lysates shows a single prominent band at 75-kDa in both healthy and prion-diseased mice brains. Image shows increased p75^NTR^ intensity (Fig. 7F) and its densitometry demonstrates significant increased expression of p75^NTR^ by 31% in prion-diseased mice brains than controls (Fig. 7G). To understand the glycosylation status of p75^NTR^, native brain lysates from both the groups were treated with PNGase F and immunoblotted. Result shows the shifting of 75-kDa band to 73-kDa upon PNGase F treatment, suggesting N-glycosylation of p75^NTR^ (Additional file 1: Fig. S2, A, middle panel). Densitometry of 75-kDa band in this experiment shows marginal increased expression of p75^NTR^ in prion-diseased mice brains than controls but not with 73-kDa p75^NTR^ isoforms (Additional file 1: Fig. S2 D, E). Nevertheless, the p75^NTR^ protein expression is marginally more in prion-diseased mice brains than controls.

### Expression profile of DR ligands in mouse prion disease

#### Alteration of TNFα and NGF expression in mouse prion disease

**Fig. 8:**
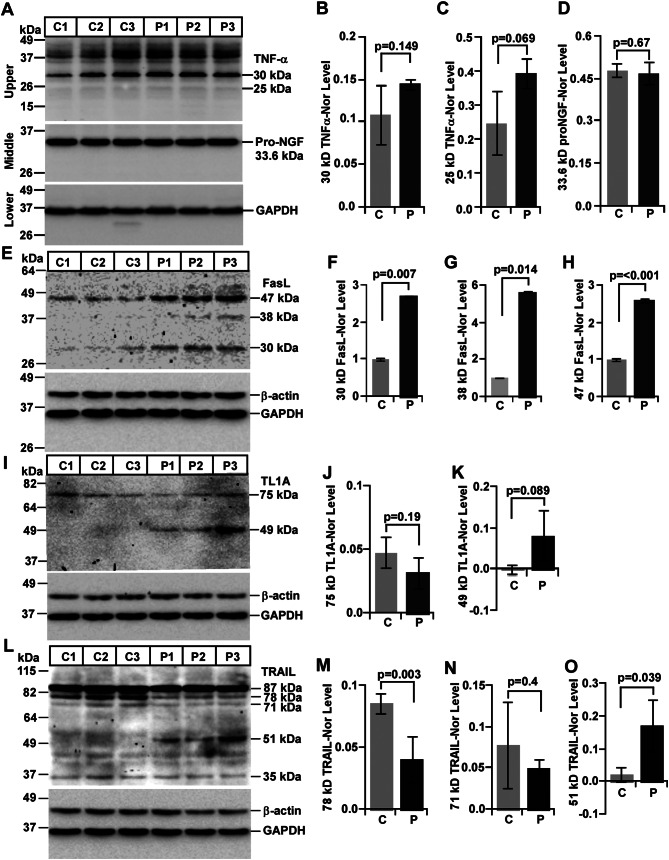
DR ligands (TNF-α, NGF, FasL, TL1A and TRAIL) are expressed differentially between control (C) and prion-diseased (P) C57BL/6J mice brains. **A-D** Unlike proNGF (A, D), the level of TNF-α (A-C) is higher with no significance in difference in prion-diseased mice brains than controls. **E-H** Levels of FasL isoforms (30, 38 and 47 kDa) are higher in prion-diseased mice brain lysates than controls. **I-K** A 49-kDa TL1A isoform is only expressed in prion-diseased mice brains but absent in controls. **L-O** Brain lysates from prion-diseased mice show higher levels of 51-kDa TRAIL isoform compared to control mice. GAPDH and β-actin were used as loading controls. Each histogram represents mean ± SD of a set of three brain lysates from control or prion-diseased mice. Two-tailed unpaired *t*-test was performed to compare the significance of difference between two groups. *P* ≤ 0.05 is considered statistically significant

TNFα is the most widely studied DR ligand in various neurodegenerative diseases, including prion diseases. To study its expression in present mouse model of prion disease, an anti-TNFα antibody was used to detect both membrane-bound (25-28-kDa) and soluble (∼ 17-kDa) TNFα isoforms. Western blot analysis of TNFα shows, marginal increased expression of 25-kDa but without any change in 30-kDa and a high molecular weight 37-kDa TNFα isoforms between control and prion groups (Fig. 8A, upper panel). In addition, soluble TNFα is not seen in the present study. Densitometry of TNFα isoforms shows a marginal overexpression of 30-kDa and 25-kDa bands (Fig. 8B, C) respectively but without significant change between prion-diseased and control mice brains.

Unlike TNFα, nerve growth factor (NGF) is one of the most common neurotrophic factors of healthy brain and the ligand of p75^NTR^. It can present both as a precursor (proNGF) and mature NGF (NGF). In order to evaluate the alteration in its level, an anti-NGF antibody developed around 192–241 amino acids of human NGF was used which can detect both proNGF and NGF. Western blot image demonstrates a single band of 33.6-kDa NGF isoforms similar to the predicted molecular mass of proNGF. Intensities of this proNGF bands remain unaltered between control and prion-diseased mice brains (Fig. 8A; middle panel). Densitometric analysis of 33.6-kDa NGF shows no significant change between control and prion groups (Fig. 8D).

#### Alteration of Fas ligand (FasL) expression in mouse prion disease

Antibody used to detect FasL was produced against 1–60 amino acids of N-terminal region of FasL, which can detect membrane-bound but not the ectodomain of FasL. Western blotting on denatured brain lysates demonstrates four bands with a molecular mass of 30-, 38-, 47-kDa FasL and its 70-90-kDa complex (data not shown). FasL bands of 30-, 38- and 47-kDa show prominent increased expression in prion-diseased mice brains than controls (Fig. 8E). Densitometry of normalized FasL bands demonstrates 30-kDa FasL isoforms level is significantly higher by 2.7 times in prion-diseased brains than controls (Fig. 8F). Similarly, the intensities of 38-kDa and 47-kDa FasL isoforms are significantly higher by 5.6 times and 2.6 times respectively between prion-diseased and control mice brains (Fig. 8G, H respectively). Taken together, the results clearly exhibit increased FasL expression in mouse prion disease.

#### Alteration of DR3 ligand (TL1A/VEGI) expression in mouse prion disease

Antibody employed to detect mouse TL1A was developed against recombinant mouse TL1A (Ile94-Leu27), which can detect both 25-28-kDa full-length (TL1A) and 19-kDa soluble (VEGI) TL1A isoforms. Anti-TL1A antibody detected a 24.18-kDa band from lung but none from normal mouse brain lysates (Additional file 1: Fig. S3) suggesting the antibody is working. Western blot image exhibits two detectable bands with a molecular mass of 49- and 75-kDa (Fig. 8I). Densitometry of 75-kDa band exhibits no change in intensity between control and prion groups (Fig. 8J), whereas, 49-kDa TL1A isoforms is restricted to all prion mice brains than any control brains (Fig. 8I). Densitometry shows approximately 100 times higher expression but with a p-value of 0.089 in prion-diseased mice brains than controls (Fig. 8K). Failure to achieve statistical significance is due to variation of TL1A expression among prion diseased brains. Nevertheless, TL1A is overexpressed in all the three prion-diseased mice brains than controls.

#### Alteration of DR5 ligand (TRAIL) expression in mouse prion disease

Anti-TRAIL antibody used in this study was produced against Pro118-Asn291 of recombinant mouse TRAIL. This antibody can detect both full-length and soluble TRAIL isoforms. Western blot image on control and prion-diseased mice brain lysates exhibits 35-, 51-, 71-, 78- and 87-kDa TRAIL isoforms (Fig. 8L). An 87-kDa TRAIL isoform, displayed marginal decreased intensity in prion diseased mice brain lysates than control brain lysates but without statistical significance (graph not shown) whereas a 78-kDa TRAIL isoform show significant decreased expression by 54% and a 71-kDa TRAIL isoform show decreased expression by 37% but without significance in prion-diseased mice brains than healthy controls (Fig. 8L-N respectively). In the present study, no band below 35-kDa is detected from any brain lysates. However, a 35-kDa TRAIL isoform is observed with marginal increase but without any statistical significance in prion-infected mice brains than controls (histograms not shown). This isoform is close to the expected TRAIL monomer (33.346-kDa). Interestingly, a 51-kDa TRAIL isoform is observed in all prion-diseased mice brains but not in any control brains (Fig. 8L). Densitometry of this TRAIL isoform shows significantly higher expression by 8-fold in prion-diseased brains than controls (Fig. 8O). Collectively, expression of TRAIL protein is increased in mouse prion disease.

### Expression of DR adaptor proteins in mouse prion disease

#### Altered expression of TRADD in mouse prion disease

**Fig. 9:**
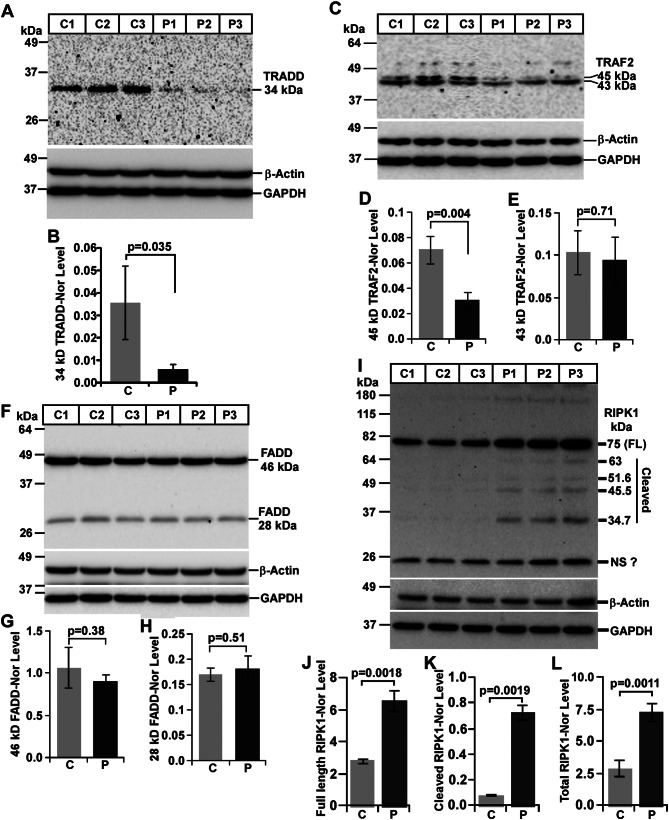
DR adaptors (TRADD, TRAF2, FADD and RIPK1) are expressed differentially between control (C) and prion-diseased (P) C57BL/6J mice brains. **A, B** Brains lysates from prion-diseased mice have significant reduced level of 34-kDa TRADD protein than control mice. **C-E** Similarly, 45-kDa TRAF2 isoform exhibit significant reduced level in brain lysates from prion-diseased mice than control mice. **F-H** Brain lysates from prion-diseased mice have equivalent level of FADD proteins, compared to control mice. **I, J, L** Prion-diseased mice brains lysates have significant higher levels of full-length (FL) and total RIPK1 protein than controls by western blot analysis. **I, K** Brain lysates from prion-diseased mice exhibit significant higher level of total cleaved (34.7-, 45.5-, 51.9-, and 63-kDa) RIPK1 products but not from control mice. GAPDH and β-actin were used as loading controls. Each histogram represents mean ± SD of a set of three brain lysates from control or prion-diseased mice. Two-tailed unpaired *t*-test was performed to compare the significance of difference between two groups. *P* ≤ 0.05 is considered statistically significant

TRADD is the common adaptor of most of the TNFR family members, which can regulate both cell survival and death. To understand the mechanism of activation of DRs, like increased expression of TNFR1 in this report, understanding the role of TRADD became warranted. The antibody used to detect TRADD was produced against a synthetic peptide surrounding cysteine 138 of human TRADD, which detects endogenous levels of human and mouse TRADD protein with a molecular mass of 34-kDa. Western blot analysis of TRADD expression exhibits a single 34-kDa band in all healthy brain lysates but barely detected in any prion brain lysates (Fig. 9A). Densitometry shows significant reduction of 34-kDa TRADD protein by 87% in prion-diseased mice brains than healthy controls (Fig. 9B). Collectively, the data suggests the inhibition of TRADD expression in this mouse prion disease.

#### Altered expression of TRAF2 in mouse prion disease

TRAF2 interacts with TRADD or RIPK1 in the context of cell survival and hence an important adaptor for the activation of TNFR family members. The antibody used to detect endogenous level of mouse TRAF2 was developed against a peptide corresponding to zinc finger TRAF type-2 region of human TRAF2. Western blot image from healthy and diseased mice brain lysates exhibits four anti-TRAF2 antibody reactive bands with molecular mass ranging from 94-, 84-, 45- and 43-kDa. A very faint 51-kDa band is also observed. All these bands are less intense in prion-diseased mice brains than controls but with maximum decrease in 45-kDa TRAF2 isoforms (Fig. 9C). Its densitometry suggests, significant decreased expression of 45-kDa TRAF2 isoforms by 57% (Fig. 9D) whereas, 99- and 84-kDa bands show 22% and 3% reduction respectively, without any statistical significance in diseased brains than controls (data not shown). Moreover, analysis of 43-kDa band intensity show 9% decreased level in diseased brains than controls (Fig. 9E) but without any significant difference. Taken together, the results represent diminished TRAF2 expression in mouse prion disease.

#### Altered expression of FADD in mouse prion disease

FADD is known to induce cell death stimulus from TNFR1, DR3, DR6 and p75NTR in a TRADD and/or RIPK1 dependent manner but from Fas and DR5 in a TRADD independent but RIPK1 dependent manner. Therefore, the study of FADD became warranted in this mouse prion disease. The antibody used to detect endogenous level of FADD was produced against a synthetic peptide corresponding to residues near the N-terminus of human FADD, which detect a 28-kDa FADD protein of both human and mouse origin. Western blot result from healthy and diseased mice brain lysates shows two bands, a 28-kDa band similar to expected molecular mass of FADD protein and a 46-kDa band. The intensities of both these bands are similar between healthy and diseased mice brains (Fig. 9F). The densitometry shows a 15% decrease in 46-kDa band intensity and merely a 5% increase in 28-kDa band intensity but without significant difference between prion-diseased and control mice brains (Fig. 9G, H respectively). Collectively, the result suggests the expression of FADD is static in mouse prion disease.

#### Altered expression of RIPK1 in mouse prion disease

Like TRADD protein, RIPK1 protein plays critical roles in both cell survival and death. However, nothing is known about its involvement in any prion disease. To study RIPK1, an anti-RIPK1 antibody developed against a synthetic peptide corresponding to residues around Leu190 of human RIPK1 was used. This antibody detects both full-length and cleaved N-terminal fragments (NTF) of RIPK1 of human and mouse origin. Western blot result shows a dominant 75-kDa band in both normal and diseased mice brains, which resembles with the molecular weight of full-length RIPK1 protein and its intensity is visibly higher in diseased mice brains than controls (Fig. 9I). The image also exhibits smaller RIPK1 63-, 51-, 45-, 35-kDa bands. Most importantly, these smaller bands are present only in prion-diseased mice brains but not in any healthy mice brain lysates (Fig. 9I). The densitometry of RIPK1 intensity normalized with housekeeping genes shows a significant increased expression of 75-kDa RIPK1 by 2.35-fold in prion mice brains than controls (Fig. 9J). In addition, the combined intensity of smaller bands is approximately 9-fold and significantly higher in prion mice brain lysate than controls (Fig. 9K). When both full-length and cleaved RIPK1 were evaluated together, the endogenous level of total RIPK1 is 2.5-fold and significantly higher in prion-diseased mice brains than control brains (Fig. 9L). Therefore, these results manifest augmented expression and proteolysis of RIPK1 in mouse prion disease.

## Discussion

This complex and pioneering study dissects the role of 16 death receptor (DR) factors using a C57BL/6J mouse model of prion disease and significantly advancing our understanding of this complex neurodegenerative disorder. Manifestations of clinical and neuropathological features in this mouse model range from abnormal gait, increased PrP^Sc^ accumulation, and pronounced astrocytosis are in line with earlier reports [6, 55–57]. Caspase-8, exclusively expressed in mice, exhibits transcriptional overexpression in prion infected C57BL/6 mice and activation in prion-infected or PrP(106–126) treated neuronal models, underscoring its pivotal role in prion pathogenesis with respect to DR pathways activation [32, 58–60]. In this study, increased level of caspase-8 might be due to the overexpression of caspase-8 gene [32]. Despite the absence of proteolytic fragments, caspase-8 activation, likely driven by dimerization, offers a critical mechanism distinct from typical proteolytic activation of executioner caspases [61–63]. Moreover, caspase-3 proteolysis underscores its position downstream of caspase-8 activation, strengthening the involvement of DR pathways in this prion disease [64]. Collectively, the data provides vital insights into the significance of DRs and their intricate interplay in disease progression.

Activation of TNFR1 by its ligand, TNFα, is one of the most commonly studied death receptor pathways. The present study meticulously examines the role of TNFR1, TNFα and their adaptor proteins within prion disease pathology, elucidating their molecular dynamics and possible functional implications. Characterized by glycosylation, oligomerization and proteolysis, TNFR1 manifests in various isoforms with molecular masses spanning 28-100-kDa [65–69]. Based on molecular weight, TNFR1 protein is profoundly glycosylated (55-kDa isoform), favoring oligomerization (∼ 100-kDa isoform). However, it is evident that, the application of PNGase F treatment to brain lysates for deglycosylation revealed obstacles in discerning mTNFR1 isoforms and oligomers, potentially hints at heat-induced aggregation of these transmembrane proteins, impeding their resolution in polyacrylamide gels [70, 71]. The present study further examined the role of soluble TNFR1 (sTNFR1) isoforms as a consequence to ectodomain shedding. The data represent a noticeable molecular weight shift (38-kDa to 35-kDa) upon deglycosylation, thereby highlighting the glycosylation of TNFR1. Glycosylation of TNFR1 favours ligand-binding affinity [72].

The augmented expression of mTNFR1 in this study, resonates with prior reports on immunocytochemistry in scrapie-infected cell lines and rodent models, substantiate our understanding of TNFR1’s role in prion disease neuropathology [30, 32]. Conversely, the level of sTNFR1 isoforms in this study, particularly the 38-kDa band and 77-88-kDa bands, presumably representing glycosylated sTNFR1 monomers and dimers respectively are reduced in this study. Soluble TNFR1 acts as a dominant negative isoform of mTNFR1, thereby decreasing TNFα bioactivity [73] but enhances neuroprotection [65].

Moreover, despite the variable expression profiles of TNFα across prion-infected experimental models it acts as a potential activator of cells with increased mTNFR1 expression [17, 33–37, 74–79]. This study hypothesizes that, even a marginal TNFα increment, in concert with alterations in sTNFR1 levels and a pronounced expression of mTNFR1 isoforms, might facilitate a pathogenic environment characterized by compromised neuroprotection and TNFα bioactivity-mediated cellular viability within prion-diseased murine brains. This principle not only deepens our comprehension of prion pathogenesis but also ushers new avenues for targeted therapeutic interventions within TNFR1 pathway.

The canonical signaling pathway originating from TNFR1 and TNFα ligation predominantly activates the NF-κB transcription factor, pivotal in inflammation, cell survival, and growth. However, if NF-κB activation is impeded, TNFR1 can induce cell death via apoptosis and necroptosis, highlighting its pleotropic function [80]. This diversity in function is largely attributed to the formation of differential DR adaptor signaling complexes at the death domain (DD). A key adaptor, TRADD, interfaces with the intracellular DD of ligated TNFR1 and other TNFR family members like DR3, DR5, DR6, and p75NTR, primarily orchestrating anti-apoptotic (NF-κB, JNK, MAPK) and alternate apoptotic pathways [81, 82]. The overexpression of TRADD can induce apoptosis and activate NF-κB through TNFR1, while its somatic knockout in B-cell lines attenuates TNFR1-mediated NF-κB activation, suggesting a predominant role in cell survival and inflammatory pathways [83, 84].

Structurally, TRADD comprises two distinct domains: the C-terminal death domain for death receptor interaction, and the N-terminal TNF receptor-associated factor 2 (TRAF2) interacting domain. Upon interaction with activated TNFR1 and death receptors like DR3, DR5, DR6, and p75NTR, TRADD complexes with TRAF2, facilitating the activation of proinflammatory NF-κB and cell survival pathways like JNK and MAPK [80]. Cells deficient in TRAF2, however, exhibit compromised TNFα-mediated JNK activation and only partial NF-κB activation, insinuating a more pronounced role for the TRADD-TRAF2 complex in cell survival rather than inflammation [85, 86]. Interestingly, the current study notes decreased TRAF2 expression in prion-diseased mice brains, potentially due to TNFα-TNFR2 ligation-mediated proteosomal degradation of TRAF2, subtly enhancing TNFR1-mediated cell death [87].

Moreover, RIPK1, another versatile DR adaptor discovered nearly three decades ago, is a 74-kDa DR signal transducer ubiquitously expressed to limit inflammation and cell death [88, 89]. Featuring a C-terminal DD, RIPK1 interacts with the DD of death receptors, TRADD, and FADD, either independently or in complex formations [80, 88, 90, 91]. The significant loss of TRADD expression in this study points towards diminished TRADD-TRAF2-RIPK1-mediated inflammatory and cell survival pathway activation, potentially heightening cell death pathways. Contrarily, RIPK1 expression is elevated in prion-diseased mice brains. It has also been shown that TRADD and RIPK1-mediates caspase activation in a redundant manner upon TNFR1 ligation [90, 92]. Therefore, the augmented RIPK1 expression in this study suggests its independent role in either promoting cell survival or cell death, detached from TNFR1 and other death receptors like DR3, DR6, and p75NTR.

Furthermore, TNFα-mediated formation of signaling complexes remains unimpeded in macrophages devoid of TRADD, which alternatively recruit RIPK1 alone or alongside TRAF2 to activate NF-κB, MAPK, and JNK pathways [80, 93]. This implies that the TRADD-TRAF2-RIPK1 axis might exhibit cell-type-specific functions, as evidenced in normal brain microglia. The observed increase in RIPK1 might associate with TNFα-TNFR1, enhancing microgliosis, a prominent neuropathology in prion disease [10]. Conversely, the deficiency in TRADD and TRAF2 might weaken neuronal and oligodendrocyte cell survival pathways while amplifying TNFα-TNFR1-RIPK1-FADD-caspase-8 mediated cell death pathways, presenting a complex landscape of cellular fate determination in prion disease pathology.

In the present study, DR3, an unexplored DR in prion disease, showcases its unique role in prion disease. This study reveals diminished DR3 levels in prion-diseased mice brains, presenting a comprehensive profile of its isoforms, including mature and glycosylated variants, alongside high molecular weight oligomers resistant to SDS denaturation [70]. The deglycosylation experiments expose the sensitivity of certain DR3 isoforms to PNGase F, hinting at their N-glycosylated nature. This intricate glycosylation pattern of DR3 is pivotal for its interaction with TL1A, a TNF-like cytokine implicated in inflammation and autoimmune diseases, yet unexplored in prion diseases [94, 95].

The further investigation into the expression patterns of TL1A, revealed the absence of both its soluble forms (∼ 22-kDa) and membrane-bound form (∼ 30-kDa without glycosylation and higher molecular weight forms based on varying degree of glycosylation) [95, 96] in normal mice brain lysates. Contrarily, a notable presence of a 49-kDa glycosylated, membrane-bound form only in prion-diseased mice brains, presents a novel, yet unique expression pattern in the pathological state. The juxtaposition of increased TL1A levels with decreased DR3 expression in prion-diseased mice brains unfolds a complex narrative, potentially explained by two hypotheses. Firstly, TL1A might induce apoptosis in DR3-expressing cells, a mechanism supported by the altered expression of key adaptor proteins resulting in a possible formation of TL1A-DR3-RIPK1-FADD-caspase-8 death inducing complex in this study [97]. Secondly, the study suggests DR3’s non-apoptotic function [70, 98]. This notion was further supported by in situ hybridization (ISH) for DR3 transcripts, showing high expression of DR3 in cortex (highest in pre-frontal cortex), hippocampus, olfactory lobe, thalamus and hind brain including cerebellum of normal C57BL/6J mice brain and its expression pattern overlaps with neurons, weakly with oligodendrocytes but not with astrocytes ISH patterns (Additional file 1: Fig. S4A). This suggests DR3 might have possibly neuroprotective role [99], a notion further reinforced by the motor deficit and associated neurological aberrations observed in DR3-deficient mice [100].

Collectively, this research elucidates the complex interplay between DR3, TL1A, and associated signaling adaptors in prion disease, shedding light on their potential roles in prion disease specific motor deficits possibly by affecting cortico-striatal neuronal pathways (Additional file 1: Fig. S4B) and thereby laying the groundwork for future explorations in prion disease mechanisms and therapeutic strategies.

In this study, DR5 (TRAIL receptor) known for its apoptotic function through interaction with TRAIL [101], unexpectedly exhibits decreased expression in prion-diseased mice brains. This includes a reduction in both the mature 36-kDa DR5 protein and its higher molecular weight glycosylated isoforms. Given the necessity of N- and O-glycosylation for DR5’s biological function, this decline poses significant implications [102, 103]. Literature suggests DR5 is primarily expressed in neuronal cells, less so in oligodendrocytes, and not in astrocytes or microglia [104]. In situ hybridization aligns DR5 expression with neuronal markers but not with astrocytic ones (Additional file 1: Fig. S5) [99]. Furthermore, the absence of DR5 in GFAP positive astroglial cells is linked to the expression of BNIP3, a transcriptional inhibitor, which mitigates DR5 expression and confers resistance to TRAIL in gliomas [105]. Physiologically, TRAIL is expressed as a ∼ 33-kDa membrane-bound TRAIL (mTRAIL) or 16-22-kDa soluble TRAIL (sTRAIL) [106–109]. sTRAIL undergo spontaneous homotrimerization with a molecular mass ranging from 63- to 66-kDa, a notion substantiated by the presence of two closely spaced 72- and 77-kDa TRAIL bands in this study [107]. This suggests lesser proteolytic cleavage of mTRAIL or increased mTRAIL isoform in mouse prion disease. Additionally, a 50-kDa TRAIL antibody reactive band seen only in prion-diseased mice brains might be the mTRAIL. Moreover, the differential ligand preferences of sTRAIL and mTRAIL for DR4 and DR5 respectively, and their roles in cancer cell proliferation, add complexity to the observed changes [29, 110].

These findings, previously unreported in prion diseases suggest possible mechanisms where, (a) increased mTRAIL, alongside altered expression of key adaptors and signaling molecules, may lead to the demise of DR5-expressing brain cells such as neurons and oligodendrocytes in a FADD dependent manner [111]. (b) Reduced DR5 expression in astrocytes and microglia possibly contribute to resistance against increased TRAIL in these cells leading to increased astrocytosis and microgliosis in prion disease [112].

Fas, a known cell death receptor, predominantly triggers apoptosis through its ligand FasL. Elevation of Fas protein in the present RML scrapie-infected C57BL/6 mice brains potentially linked to the increased mRNA levels while differing from other murine scrapie models [32, 39, 42]. Enhanced levels of Fas protein bands (38 to 52-kDa) observed in all prion-diseased mouse brain lysates, independent of SDS presence, align with those identified in BJAB cells. This increase in molecular weight is likely due to differential glycosylation at the Fas protein’s N-linked glycosylation sites [113]. Treatment with N-ethylamide notably increased glycosylated Fas isoforms (48-51-kDa) in rat cerebral cortical membranes [114] and palmitoylated Fas proteins typically appear between 45- and 55-kDa [115], resembling the 52-kDa band in this study. PNGase F deglycosylation indicated that the 52-kDa band is likely palmitoylated, consistent with previous findings [115], whereas the diminished intensity of the 48-, 42-, and 38-kDa bands post-treatment suggests N-glycosylation. Both modifications are known to stabilize receptor aggregates initiating apoptotic signalling [114, 116]. A 94-kDa band detected using a C-terminus specific anti-Fas antibody may represent C-terminal Fas fragment aggregates, with its reduced prevalence in prion-diseased mouse brains indicating suppressed formation of soluble Fas, which typically inhibits cell death by sequestering FasL [113]. FasL is mainly expressed in the liver, heart, thymus, and ovary, with minimal expression in the spleen and brain of mice [117]. Control mouse brains in this study showed low levels of 30- and 48-kDa FasL isoforms, whereas prion-diseased brains exhibited an additional 37-kDa band. Given mouse native FasL’s size is of 31-kDa and potential for glycosylation, the 30-kDa band is likely unglycosylated, while the 37- and 48-kDa bands are glycosylated membrane-bound FasL isoforms [118]. The absence of a 26-kDa soluble FasL isoform [118] suggests exclusive expression of membrane-bound FasL in prion-diseased mouse brains, known for its higher cytotoxicity than its soluble counterpart [119, 120].

Notably, all membrane-bound FasL isoforms are more prevalent in prion-diseased mouse brains than in controls, contrasting reports of variable FasL expression in other prion diseases [41, 42] and suggesting a unique pathophysiological mechanism in this mouse prion disease model. The interaction of Fas with FasL typically induces apoptosis via FADD and caspase-8 [28]. Consequently, the increase in membrane-bound and glycosylated Fas isoforms, coupled with decreased soluble Fas levels and elevated membrane-bound FasL isoforms, potentially skews cell fate towards apoptosis in this mouse prion disease model. Moreover, Fas also incites proinflammatory responses and inhibits apoptosis in macrophages (microglia in the brain) by engaging RIPK1 independently of TRADD [23]. The upregulated Fas, FasL, and RIPK1, alongside reduced TRADD levels, may account for the pronounced microgliosis observed in this prion disease mouse model.

Death receptor 6 (DR6), a lesser-explored member of the TNFR superfamily, considered as an orphan DR due to the lack of a specific ligand [121, 122]. In situ hybridization analysis of normal C57BL/6J mouse brains reveals extensive DR6 expression throughout the brain (Allen Brain Mouse Atlas: mouse.brain-map.org/experiment/show/68,666,535), indicating its potential role in the maintenance of mature brain cells rather than serving as a death stimulator [99]. The identification of 74- and 89-kDa DR6 isoforms in SDS-containing brain homogenates, alongside a 94-kDa isoform in non-SDS homogenates, points to the existence of distinct glycosylated DR6 forms. Notably, PNGase F treatment shifts the 94-kDa band to 80-kDa, suggesting multiple N-glycosylation of the DR6 protein. However, the absence of unglycosylated DR6 isoforms (68-72-kDa) and 80-kDa DR6 band following PNGase F treatment suggest additional unexplored glycosylation modifications, such as O-glycosylation at several threonine and serine residues and palmitoylation at the cysteine 368 residue [123]. The intensity of the 94-kDa band in lysates without SDS decreases significantly, while the 80-kDa band exhibits a declining trend in the terminal phase of prion-diseased mouse brains, suggesting a potential impairment in DR6 function, like axon pruning and neuronal plasticity rearrangements in adult mice brains [124, 125]. Additionally, DR6 is implicated in complex formation with p75NTR, further highlighting its multifaceted role in disease pathology [122].

The neurotrophin receptor p75^NTR^ (presented as a 75-kDa protein) with a variety of neuronal functions [126, 127], exhibited its overexpression in prion-diseased mice brain lysates with SDS similar to the earlier reports [43, 44, 128, 129]. Moreover, shifting of 75-kDa to ∼ 73-kDa post PNGase F treatment indicates its N-glycosylation but failure to attend theoretical molecular mass (40-kDa) suggests additional and unexamined glycosylations like palmitoylation [130] and *O*-glycosylation [131] of p75^NTR^. Invariable expression of tropomyosin-related kinase A (TrkA) and sortilin (Additional file 1: Fig. S6), the co-receptors of p75^NTR^ for cell survival and death respectively, suggest their insignificant role in prion disease, so also with proNGF/NGF, the genuine ligand of p75^NTR^ [132]. On the other hand, p75^NTR^ either independently or partnering with DR6 interacts with β-amyloid peptides to induce neuronal cell deaths in experimental models [122, 133–135]. Similarly, β-sheet rich PrP(106–126) peptides trigger neuronal damage by interacting with p75^NTR^ [129]. Therefore, abundant levels of β-sheet rich PrP^Sc^, decreased TRADD and TRAF2 level, and increased RIPK1, caspase-8 level along with sustained FADD expression might activate p75^NTR^ either independently or in complex with DR6 to trigger neuronal damage over neuroprotection, highlighting its multifaceted role in pathophysiological mechanism in this mouse prion disease model.

Finally, RIPK1, the only adaptor protein that interacts with all DD containing death receptors, and other adaptor proteins, TRADD, TRAF2 and FADD making its position central to all kinds of death receptor functions [80, 88, 90, 91]. Along with elevated expression of its full-length form (75-kDa) as discussed previously, this study meticulously examines the proteolysis of RIPK1, a potent signature of cell apoptosis. Based on the epitope of the anti-RIPK1 antibody used, a 35-kDa N-terminal fragment (NTF) identified in this study, parallels with the 39-kDa C-terminal fragment (CTF) of RIPK1 from caspase-8 cleavage [136, 137]. Correspondingly, its 25- and 45-kDa NTFs from this study authenticates the 30- and 50-kDa CTF of RIPK1 from cathepsins cleavage [138] and its 51-kDa NTF in this study validates the 25 kDa CTF of RIPK1 from HtrA2/Omi, a serine protease proteolysis [139]. Proteolysis of RIPK1 either by caspase-8, cathepsins or HtrA2/Omi losses its cell survival and necroptosis activity, conversely promote apoptosis [136–139]. Increased levels of caspase-8 in this study and cathepsins in other experimental prion study [140] mirror the augmented presentation of cleaved RIPK1 fragments, restricted to prion-diseased mice brain elucidating a paradigm shift from cell survival and necroptosis to cellular apoptosis.

It is important to acknowledge that, the report describes the data obtained from one animal model of prion disease with a sample size of three independent mice in each group, which limits robust statistical analysis. These limitations warrant future experiments involving other mouse and prion strains with an increased sample size. Additionally, there are several limitations to the methodology used in this study. For example, whether altered expression of death receptor factors at protein level are the consequence of altered gene expression is not studied in this study. Such RNA experiments can be incorporated in future experimental model using Q-RT-PCR. Moreover, no functional outcomes were measured for neurodegeneration in the current study. Therefore, further validation using alternative methods and additional prion model systems in future studies to solidify the direct association and functional significance of these altered death receptor factor proteins is indispensable to elucidate the neuropathological conditions of prion disease. Despite these limitations, the results from this pioneering research employing a fully penetrant and inbreed mouse model of prion disease not only elucidate multiple death receptor pathways operating both at pleotropic and polygenic level but also furthers our understanding on prion disease related neuropathology.

## Conclusions

**Fig. 10:**
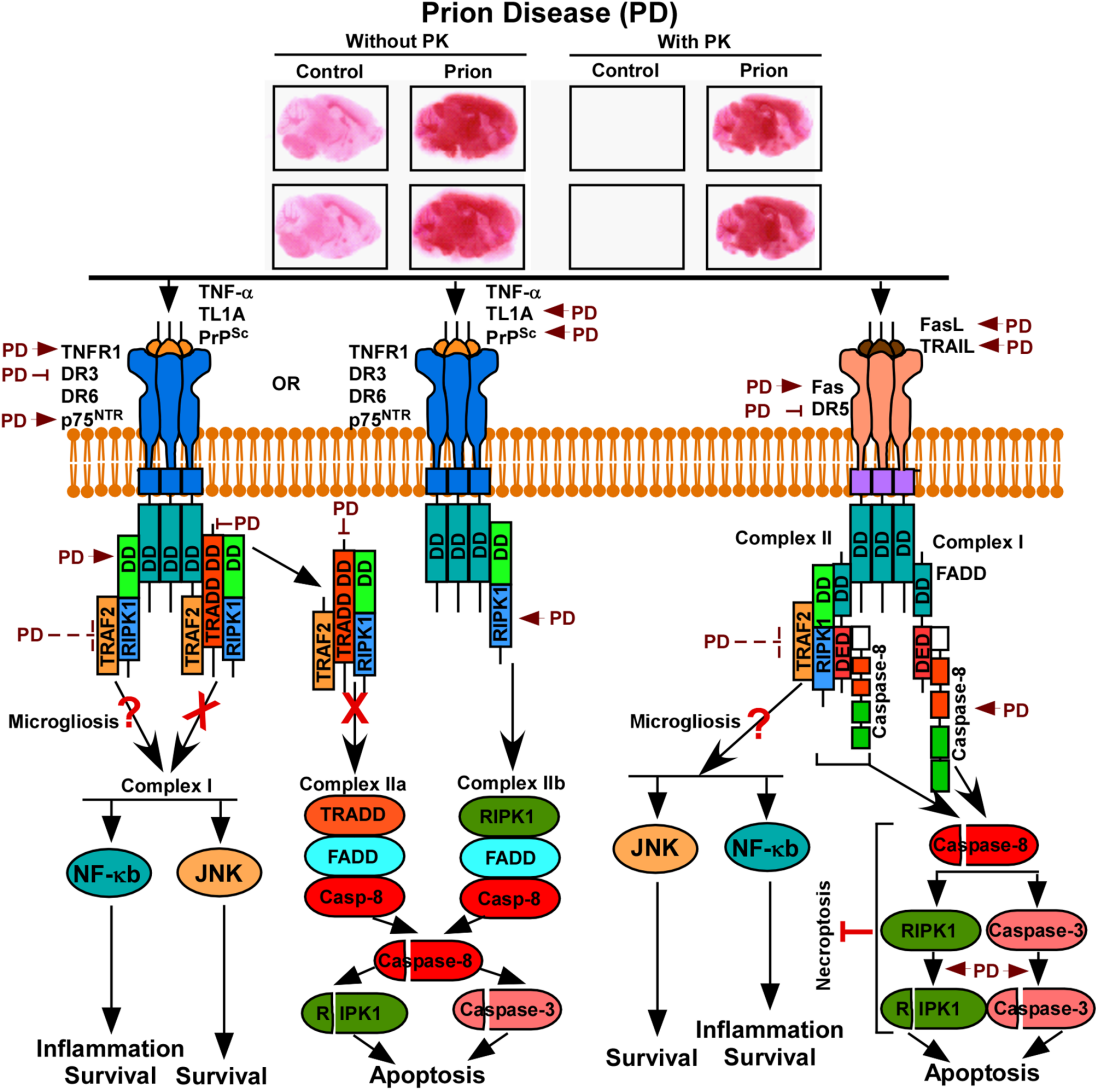
Schematic interpretation of probable mechanisms regulated by major death receptors (DRs) pathways, based on altered expression pattern of DRs, their ligands and adaptors in C57BL/6J mouse prion disease. Upon binding to their specific ligands, TNFR1, DR3, DR6, and p75NTR activate primarily NFκB, MAPK and JNK-mediated proinflammatory and antiapoptotic pathways by recruiting TRADD-RIPK1-TRAF2 or TRADD-RIPK1 or RIPK1-TRAF2 adaptors to form complex-I. Similar complexes can be formed from TRAIL-DR5 ligation. Reduced expression of TRADD and TRAF2 but increased proteolysis of RIPK1 clearly suggests the inhibition of complex-I and -IIa formation, upon the ligation of these DRs. Thus, increased expression of TNFα, TL1A, PrP^Sc^, full-length RIPK1 along with sustained level of FADD clearly suggest the formation of complex-IIb from TNFR1, DR3 and DR6/p75NTR respectively by forming complex with RIPK1-FADD-caspase-8. Ligation of Fas and DR5 by their specific ligands, mediate mainly apoptotic pathway by forming complex with either FADD-caspase-8 or RIPK1-FADD-caspase-8. Thus, higher levels of FasL and TRAIL in this study suggest activation of apoptosis. Furthermore, increased cleavage of RIPK1 and caspase-3 indicate caspase-8 activation, which are established molecular markers of apoptosis and inhibitors of necroptosis. Increased expression of FasL, Fas and RIPK1 suggests proinflammatory and antiapoptotic response from macrophages (microglia in brain). Similar responses can be achieved from increased expression of TNFR1, RIPK1 along with sustained levels of TNFα in microglia in a TRADD independent manner. Finally, reduced expression of DR5 along with increased expression of TRAIL can be considered as molecular signature of astrocytosis in mouse prion disease. An arrow ($\rightarrow$) represents either increased expression of a DR factor or activation of a specific pathway. A cross road ($\dashv$) suggests either reduced expression of a DR factor or inhibition of a specific pathway. Any factor without any an arrow or cross-road is considered as static expression in prion disease. Finally, the inhibition of a pathway resulting from prion disease is overlayed by a cross in red color (X)

The study concludes with the analysis of 16 death receptor factors, uncovering critical insights into the neuropathological mechanisms of prion disease, represented schematically in Fig. 10 and additional file 3: presentation S1. The findings suggest possible inhibition of cell survival pathways (TNFR1, DR3, DR6, p75^NTR^, partially DR5 and Fas) due to marked reduction in TRADD and TRAF2 expression, the vulnerability of DR3 and DR5 expressing cells indicated by TL1A and TRAIL overexpression, and steady FADD levels coupled with elevated FasL, Fas, TRAIL, and caspase-8 pointing towards apoptotic cell demise. Enhanced RIPK1 proteolysis with caspase-8 cleavage signature confirms apoptosis, while high levels of RIPK1 and caspase-8 alongside constant FADD may induce apoptosis through Fas, DR5, TNFR1, DR3 and p75^NTR^ ligation. Overexpressed TNFR1, Fas, FasL, and stable TNFα levels combined with reduced DR5 might promote astrocytosis and microgliosis in a TRADD-independent but RIPK1-dependent manner. Novel glycosylated and oligomeric TNFR1 and FAS isoforms imply enhanced ligand binding, and diminished monomeric, oligomeric, and glycosylated soluble TNFR1 and Fas isoforms indicate impaired cell protection, contributing to cell death in prion diseases. Lastly, neurodegeneration in prion diseases appears to be driven not by a single death receptor pathway but by multifactorial, multi-receptor-mediated cell death processes, as evidenced in this mouse model of prion disease. Despite the limitations as mentioned earlier, the present study narrates a complex network of death receptors, their ligand and adaptor proteins elaborately intertwined into the multifarious nature of prion disease neuropathology, thereby laying a robust foundation for future explorations into their functional association with prion-induced neurodegeneration and potential avenues for targeted therapeutic interventions. Moreover, findings from this research may help in designing and developing decoys for TL1A, TRAIL and FasL to restore DR3, DR5 and Fas pathways respectively in experimental prion diseases. Similarly restoring RIPK1, TRADD and TRAF2 expression would be direct clinical implications on prion diseases. Notably, deep brain stimulation of cortico-striatal pathway may restore motor deficit in prion patients, which would be another clinical implication of the data obtained from the present mouse model of prion disease.

### Electronic supplementary material

Below is the link to the electronic supplementary material.

Supplementary Material 1

Supplementary Material 2

Supplementary Material 3

Supplementary Material 4

Supplementary Material 5

Supplementary Material 6

Supplementary Material 7

Supplementary Material 8

Supplementary Material 9

Supplementary Material 10

## Data Availability

The datasets analysed during the current study are available from the corresponding author upon request.

## Abbreviations

DD: Death domain
DR: Death receptor
TNFα: Tumor necrosis factor alpha
TNFR1: Tumor necrosis factor receptor 1
Fas: FS7-associated surface antigen
TL1A: TNF like ligand 1 A
TRAIL: TNF-related apoptosis-inducing ligand
TRADD: TNFR1-associated death domain
TRAF2: TNFR-associated factor 2
FADD: Fas-associated death domain
RIPK1: Receptor interacting protein kinase 1
NGF: Nerve growth factor
P75NTR: The neurotrophin receptor p75
GFAP: Glial fibrillar acidic protein
GAPDH: Glyceraldehyde-3-phosphate dehydrogenase

## References

[1] Brown AR, Rebus S, McKimmie CS, Robertson K, Williams A, Fazakerley JK (2005). Gene expression profiling of the preclinical scrapie-infected hippocampus. Biochem Biophys Res Commun.

[2] Prusiner SB (1996). Molecular biology and pathogenesis of prion diseases. Trends Biochem Sci.

[3] Prusiner SB, Prions (1998). Proc Natl Acad Sci U S A.

[4] Xiang W, Windl O, Wunsch G, Dugas M, Kohlmann A, Dierkes N, Westner IM, Kretzschmar HA (2004). Identification of differentially expressed genes in scrapie-infected mouse brains by using global gene expression technology. J Virol.

[5] Prusiner SB (1982). Novel proteinaceous infectious particles cause scrapie. Science.

[6] DeArmond SJ, Prusiner SB (1995). Prion protein transgenes and the neuropathology in prion diseases. Brain Pathol.

[7] Marella M, Chabry J (2004). Neurons and astrocytes respond to prion infection by inducing microglia recruitment. J Neurosci.

[8] Brandner S, Isenmann S, Raeber A, Fischer M, Sailer A, Kobayashi Y, Marino S, Weissmann C, Aguzzi A (1996). Normal host prion protein necessary for scrapie-induced neurotoxicity. Nature.

[9] Yusa S, Oliveira-Martins JB, Sugita-Konishi Y, Kikuchi Y (2012). Cellular prion protein: from physiology to pathology. Viruses.

[10] Prusiner SB (1994). Biology and genetics of prion diseases. Annu Rev Microbiol.

[11] Sikorska B (2004). Mechanisms of neuronal death in transmissible spongiform encephalopathies. Folia Neuropathol.

[12] Liberski PP, Brown DR, Sikorska B, Caughey B, Brown P (2008). Cell death and autophagy in prion diseases (transmissible spongiform encephalopathies). Folia Neuropathol.

[13] Lopez-Perez O, Toivonen JM, Otero A, Solanas L, Zaragoza P, Badiola JJ, Osta R, Bolea R, Martin-Burriel I (2020). Impairment of autophagy in scrapie-infected transgenic mice at the clinical stage. Lab Invest.

[14] Mammadova N, West Greenlee MH, Moore SJ, Sakaguchi DS, Greenlee JJ (2020). Experimental study using multiple strains of prion disease in cattle reveals an inverse relationship between incubation time and misfolded prion Accumulation, Neuroinflammation, and Autophagy. Am J Pathol.

[15] Shah SZA, Zhao D, Hussain T, Sabir N, Mangi MH, Yang L (2018). p62-Keap1-NRF2-ARE pathway: a contentious player for selective targeting of Autophagy, oxidative stress and mitochondrial dysfunction in Prion diseases. Front Mol Neurosci.

[16] Thellung S, Corsaro A, Dellacasagrande I, Nizzari M, Zambito M, Florio T (2022). Proteostasis unbalance in prion diseases: mechanisms of neurodegeneration and therapeutic targets. Front Neurosci.

[17] Hong JM, Moon JH, Oh YM, Park SY, Calcineurin (2021). Calcium-dependent serine-threonine phosphatase activation by prion peptide 106–126 enhances nuclear factor-kappab-linked proinflammatory response through Autophagy Pathway. ACS Chem Neurosci.

[18] Liberski PP, Gajos A, Bogucki A (2017). Robust autophagy in optic nerves of experimental Creutzfeldt-Jakob disease and Gerstmann-Straussler-Scheinker disease. Folia Neuropathol.

[19] Ma Y, Shi Q, Xiao K, Wang J, Chen C, Gao LP, Gao C, Dong XP (2019). Stimulations of the Culture Medium of Activated Microglia and TNF-Alpha on a scrapie-infected cell line decrease the cell viability and induce marked necroptosis that also occurs in the brains from the patients of Human Prion diseases. ACS Chem Neurosci.

[20] Chiesa R, Piccardo P, Biasini E, Ghetti B, Harris DA (2008). Aggregated, wild-type prion protein causes neurological dysfunction and synaptic abnormalities. J Neurosci.

[21] Harris DA, True HL (2006). New insights into prion structure and toxicity. Neuron.

[22] Lavrik I, Golks A, Krammer PH (2005). Death receptor signaling. J Cell Sci.

[23] Wilson NS, Dixit V, Ashkenazi A (2009). Death receptor signal transducers: nodes of coordination in immune signaling networks. Nat Immunol.

[24] Green DR (2022). The death receptor pathway of apoptosis. Cold Spring Harb Perspect Biol.

[25] Haase G, Pettmann B, Raoul C, Henderson CE (2008). Signaling by death receptors in the nervous system. Curr Opin Neurobiol.

[26] Vial J, Royet A, Cassier P, Tortereau A, Dinvaut S, Maillet D, Gratadou-Hupon L, Creveaux M, Sadier A, Tondeur G (2019). The ectodysplasin receptor EDAR acts as a tumor suppressor in melanoma by conditionally inducing cell death. Cell Death Differ.

[27] Micheau O, Tschopp J (2003). Induction of TNF receptor I-mediated apoptosis via two sequential signaling complexes. Cell.

[28] Peter ME, Krammer PH (2003). The CD95(APO-1/Fas) DISC and beyond. Cell Death Differ.

[29] Kimberley FC, Screaton GR (2004). Following a TRAIL: update on a ligand and its five receptors. Cell Res.

[30] Pietri M, Dakowski C, Hannaoui S, Alleaume-Butaux A, Hernandez-Rapp J, Ragagnin A, Mouillet-Richard S, Haik S, Bailly Y, Peyrin JM (2013). PDK1 decreases TACE-mediated alpha-secretase activity and promotes disease progression in prion and Alzheimer’s diseases. Nat Med.

[31] Ragagnin A, Ezpeleta J, Guillemain A, Boudet-Devaud F, Haeberle AM, Demais V, Vidal C, Demuth S, Beringue V, Kellermann O (2018). Cerebellar compartmentation of prion pathogenesis. Brain Pathol.

[32] Stobart MJ, Parchaliuk D, Simon SL, Lemaistre J, Lazar J, Rubenstein R, Knox JD (2007). Differential expression of interferon responsive genes in rodent models of transmissible spongiform encephalopathy disease. Mol Neurodegener.

[33] Campbell IL, Eddleston M, Kemper P, Oldstone MB, Hobbs MV (1994). Activation of cerebral cytokine gene expression and its correlation with onset of reactive astrocyte and acute-phase response gene expression in scrapie. J Virol.

[34] Cunningham C, Campion S, Lunnon K, Murray CL, Woods JF, Deacon RM, Rawlins JN, Perry VH (2009). Systemic inflammation induces acute behavioral and cognitive changes and accelerates neurodegenerative disease. Biol Psychiatry.

[35] Kordek R, Nerurkar VR, Liberski PP, Isaacson S, Yanagihara R, Gajdusek DC (1996). Heightened expression of tumor necrosis factor alpha, interleukin 1 alpha, and glial fibrillary acidic protein in experimental Creutzfeldt-Jakob disease in mice. Proc Natl Acad Sci U S A.

[36] Stoeck K, Bodemer M, Zerr I (2006). Pro- and anti-inflammatory cytokines in the CSF of patients with Creutzfeldt-Jakob disease. J Neuroimmunol.

[37] Thackray AM, McKenzie AN, Klein MA, Lauder A, Bujdoso R (2004). Accelerated prion disease in the absence of interleukin-10. J Virol.

[38] Xie WL, Shi Q, Zhang J, Zhang BY, Gong HS, Guo Y, Wang SB, Xu Y, Wang K, Chen C (2013). Abnormal activation of microglia accompanied with disrupted CX3CR1/CX3CL1 pathway in the brains of the hamsters infected with scrapie agent 263K. J Mol Neurosci.

[39] Field R, Campion S, Warren C, Murray C, Cunningham C (2010). Systemic challenge with the TLR3 agonist poly I:C induces amplified IFNalpha/beta and IL-1beta responses in the diseased brain and exacerbates chronic neurodegeneration. Brain Behav Immun.

[40] Jamieson E, Jeffrey M, Ironside JW, Fraser JR (2001). Activation of Fas and caspase 3 precedes PrP accumulation in 87V scrapie. NeuroReport.

[41] Puig B, Ferrer I (2001). Cell death signaling in the cerebellum in Creutzfeldt-Jakob disease. Acta Neuropathol.

[42] Siso S, Puig B, Varea R, Vidal E, Acin C, Prinz M, Montrasio F, Badiola J, Aguzzi A, Pumarola M, Ferrer I (2002). Abnormal synaptic protein expression and cell death in murine scrapie. Acta Neuropathol.

[43] Barrio T, Vidal E, Betancor M, Otero A, Martin-Burriel I, Monzon M, Monleon E, Pumarola M, Badiola JJ, Bolea R (2021). Evidence of p75 neurotrophin receptor involvement in the Central Nervous System Pathogenesis of Classical Scrapie in Sheep and a transgenic mouse model. Int J Mol Sci.

[44] Marco-Salazar P, Marquez M, Fondevila D, Rabanal RM, Torres JM, Pumarola M, Vidal E (2014). Mapping of neurotrophins and their receptors in the adult mouse brain and their role in the pathogenesis of a transgenic murine model of bovine spongiform encephalopathy. J Comp Pathol.

[45] Wang TT, Tian C, Sun J, Wang H, Zhang BY, Chen C, Wang J, Xiao K, Chen LN, Lv Y (2016). Down-regulation of brain-derived neurotrophic factor and its signaling components in the brain tissues of scrapie experimental animals. Int J Biochem Cell Biol.

[46] Albrecht D, Garcia L, Cartier L, Kettlun AM, Vergara C, Collados L, Valenzuela MA (2006). Trophic factors in cerebrospinal fluid and spinal cord of patients with tropical spastic paraparesis, HIV, and Creutzfeldt-Jakob disease. AIDS Res Hum Retroviruses.

[47] Hu C, Chen C, Chen J, Xiao K, Wang J, Shi Q, Ma Y, Gao LP, Wu YZ, Liu L (2021). The low levels of nerve growth factor and its upstream regulatory kinases in prion infection is reversed by resveratrol. Neurosci Res.

[48] Carlson GA, Kingsbury DT, Goodman PA, Coleman S, Marshall ST, DeArmond S, Westaway D, Prusiner SB (1986). Linkage of prion protein and scrapie incubation time genes. Cell.

[49] Giri RK, Young R, Pitstick R, DeArmond SJ, Prusiner SB, Carlson GA (2006). Prion infection of mouse neurospheres. Proc Natl Acad Sci U S A.

[50] Chandler RL (1961). Encephalopathy in mice produced by inoculation with scrapie brain material. Lancet.

[51] Mastrianni JA, Nixon R, Layzer R, Telling GC, Han D, DeArmond SJ, Prusiner SB (1999). Prion protein conformation in a patient with sporadic fatal insomnia. N Engl J Med.

[52] Piro JR, Harris BT, Nishina K, Soto C, Morales R, Rees JR, Supattapone S (2009). Prion protein glycosylation is not required for strain-specific neurotropism. J Virol.

[53] Ghate PS, Sidhar H, Carlson GA, Giri RK (2014). Development of a novel cellular model of Alzheimer’s disease utilizing neurosphere cultures derived from B6C3-Tg(APPswe,PSEN1dE9)85Dbo/J embryonic mouse brain. Springerplus.

[54] Boatright KM, Salvesen GS (2003). Mechanisms of caspase activation. Curr Opin Cell Biol.

[55] Carlson GA, Ebeling C, Yang SL, Telling G, Torchia M, Groth D, Westaway D, DeArmond SJ, Prusiner SB (1994). Prion isolate specified allotypic interactions between the cellular and scrapie prion proteins in congenic and transgenic mice. Proc Natl Acad Sci U S A.

[56] Tatzelt J, Groth DF, Torchia M, Prusiner SB, DeArmond SJ (1999). Kinetics of prion protein accumulation in the CNS of mice with experimental scrapie. J Neuropathol Exp Neurol.

[57] Westaway D, DeArmond SJ, Cayetano-Canlas J, Groth D, Foster D, Yang SL, Torchia M, Carlson GA, Prusiner SB (1994). Degeneration of skeletal muscle, peripheral nerves, and the central nervous system in transgenic mice overexpressing wild-type prion proteins. Cell.

[58] Kristiansen M, Messenger MJ, Klohn PC, Brandner S, Wadsworth JD, Collinge J, Tabrizi SJ (2005). Disease-related prion protein forms aggresomes in neuronal cells leading to caspase activation and apoptosis. J Biol Chem.

[59] Sahu U, Sidhar H, Ghate PS, Advirao GM, Raghavan SC, Giri RK (2013). A Novel Anticancer Agent, 8-Methoxypyrimido[4’,5’:4,5]thieno(2,3-b) Quinoline-4(3H)-One induces Neuro 2a Neuroblastoma Cell Death through p53-Dependent, caspase-dependent and -independent apoptotic pathways. PLoS ONE.

[60] White AR, Guirguis R, Brazier MW, Jobling MF, Hill AF, Beyreuther K, Barrow CJ, Masters CL, Collins SJ, Cappai R (2001). Sublethal concentrations of prion peptide PrP106-126 or the amyloid beta peptide of Alzheimer’s disease activates expression of proapoptotic markers in primary cortical neurons. Neurobiol Dis.

[61] Boatright KM, Renatus M, Scott FL, Sperandio S, Shin H, Pedersen IM, Ricci JE, Edris WA, Sutherlin DP, Green DR, Salvesen GS (2003). A unified model for apical caspase activation. Mol Cell.

[62] Donepudi M, Mac Sweeney A, Briand C, Grutter MG (2003). Insights into the regulatory mechanism for caspase-8 activation. Mol Cell.

[63] Sohn D, Schulze-Osthoff K, Janicke RU (2005). Caspase-8 can be activated by interchain proteolysis without receptor-triggered dimerization during drug-induced apoptosis. J Biol Chem.

[64] Stennicke HR, Jurgensmeier JM, Shin H, Deveraux Q, Wolf BB, Yang X, Zhou Q, Ellerby HM, Ellerby LM, Bredesen D (1998). Pro-caspase-3 is a major physiologic target of caspase-8. J Biol Chem.

[65] Bartsch JW, Wildeboer D, Koller G, Naus S, Rittger A, Moss ML, Minai Y, Jockusch H (2010). Tumor necrosis factor-alpha (TNF-alpha) regulates shedding of TNF-alpha receptor 1 by the metalloprotease-disintegrin ADAM8: evidence for a protease-regulated feedback loop in neuroprotection. J Neurosci.

[66] Chhibber-Goel J, Coleman-Vaughan C, Agrawal V, Sawhney N, Hickey E, Powell JC, McCarthy JV (2016). Gamma-secretase activity is required for regulated intramembrane proteolysis of Tumor Necrosis factor (TNF) receptor 1 and TNF-mediated pro-apoptotic signaling. J Biol Chem.

[67] Corti A, Merli S, Bagnasco L, D’Ambrosio F, Marino M, Cassani G (1995). Identification of two forms (31–33 and 48 kD) of the urinary soluble p55 tumor necrosis factor receptor that are differentially N- and O-glycosylated. J Interferon Cytokine Res.

[68] Cui X, Hawari F, Alsaaty S, Lawrence M, Combs CA, Geng W, Rouhani FN, Miskinis D, Levine SJ (2002). Identification of ARTS-1 as a novel TNFR1-binding protein that promotes TNFR1 ectodomain shedding. J Clin Invest.

[69] Hawari FI, Rouhani FN, Cui X, Yu ZX, Buckley C, Kaler M, Levine SJ (2004). Release of full-length 55-kDa TNF receptor 1 in exosome-like vesicles: a mechanism for generation of soluble cytokine receptors. Proc Natl Acad Sci U S A.

[70] Gout S, Morin C, Houle F, Huot J (2006). Death receptor-3, a new E-Selectin counter-receptor that confers migration and survival advantages to colon carcinoma cells by triggering p38 and ERK MAPK activation. Cancer Res.

[71] Tsuji Y (2020). Transmembrane protein Western blotting: impact of sample preparation on detection of SLC11A2 (DMT1) and SLC40A1 (ferroportin). PLoS ONE.

[72] Han L, Zhang D, Tao T, Sun X, Liu X, Zhu G, Xu Z, Zhu L, Zhang Y, Liu W (2015). The role of N-glycan modification of TNFR1 in inflammatory microglia activation. Glycoconj J.

[73] Nophar Y, Kemper O, Brakebusch C, Englemann H, Zwang R, Aderka D, Holtmann H, Wallach D (1990). Soluble forms of tumor necrosis factor receptors (TNF-Rs). The cDNA for the type I TNF-R, cloned using amino acid sequence data of its soluble form, encodes both the cell surface and a soluble form of the receptor. EMBO J.

[74] Brown AR, Webb J, Rebus S, Walker R, Williams A, Fazakerley JK (2003). Inducible cytokine gene expression in the brain in the ME7/CV mouse model of scrapie is highly restricted, is at a strikingly low level relative to the degree of gliosis and occurs only late in disease. J Gen Virol.

[75] Cunningham C, Boche D, Perry VH (2002). Transforming growth factor beta1, the dominant cytokine in murine prion disease: influence on inflammatory cytokine synthesis and alteration of vascular extracellular matrix. Neuropathol Appl Neurobiol.

[76] Kim JI, Ju WK, Choi JH, Choi E, Carp RI, Wisniewski HM, Kim YS (1999). Expression of cytokine genes and increased nuclear factor-kappa B activity in the brains of scrapie-infected mice. Brain Res Mol Brain Res.

[77] Kouadir M, Yang L, Tan R, Shi F, Lu Y, Zhang S, Yin X, Zhou X, Zhao D (2012). CD36 participates in PrP(106–126)-induced activation of microglia. PLoS ONE.

[78] Srivastava S, Katorcha E, Makarava N, Barrett JP, Loane DJ, Baskakov IV (2018). Inflammatory response of microglia to prions is controlled by sialylation of PrP(sc). Sci Rep.

[79] Walsh DT, Betmouni S, Perry VH (2001). Absence of detectable IL-1beta production in murine prion disease: a model of chronic neurodegeneration. J Neuropathol Exp Neurol.

[80] Pobezinskaya YL, Liu Z (2012). The role of TRADD in death receptor signaling. Cell Cycle.

[81] Chen Y, Gu Y, Xiong X, Zheng Y, Liu X, Wang W, Meng G (2022). Roles of the adaptor protein tumor necrosis factor receptor type 1-associated death domain protein (TRADD) in human diseases. Biomed Pharmacother.

[82] Hsu H, Shu HB, Pan MG, Goeddel DV (1996). TRADD-TRAF2 and TRADD-FADD interactions define two distinct TNF receptor 1 signal transduction pathways. Cell.

[83] Hsu H, Xiong J, Goeddel DV (1995). The TNF receptor 1-associated protein TRADD signals cell death and NF-kappa B activation. Cell.

[84] Schneider F, Neugebauer J, Griese J, Liefold N, Kutz H, Briseno C, Kieser A (2008). The viral oncoprotein LMP1 exploits TRADD for signaling by masking its apoptotic activity. PLoS Biol.

[85] Yeh WC, Shahinian A, Speiser D, Kraunus J, Billia F, Wakeham A, de la Pompa JL, Ferrick D, Hum B, Iscove N (1997). Early lethality, functional NF-kappaB activation, and increased sensitivity to TNF-induced cell death in TRAF2-deficient mice. Immunity.

[86] Zhang L, Blackwell K, Workman LM, Gibson-Corley KN, Olivier AK, Bishop GA, Habelhah H (2016). TRAF2 exerts opposing effects on basal and TNFalpha-induced activation of the classic IKK complex in hematopoietic cells in mice. J Cell Sci.

[87] Li X, Yang Y, Ashwell JD (2002). TNF-RII and c-IAP1 mediate ubiquitination and degradation of TRAF2. Nature.

[88] Newton K (2020). Multitasking kinase RIPK1 regulates cell death and inflammation. Cold Spring Harb Perspect Biol.

[89] Stanger BZ, Leder P, Lee TH, Kim E, Seed B (1995). RIP. A novel protein containing a death domain that interacts with Fas/APO-1 (CD95) in yeast and causes cell death. Cell.

[90] Fullsack S, Rosenthal A, Wajant H, Siegmund D (2019). Redundant and receptor-specific activities of TRADD, RIPK1 and FADD in death receptor signaling. Cell Death Dis.

[91] Wajant H, Siegmund D (2019). TNFR1 and TNFR2 in the control of the Life and Death Balance of macrophages. Front Cell Dev Biol.

[92] Pobezinskaya YL, Kim YS, Choksi S, Morgan MJ, Li T, Liu C, Liu Z (2008). The function of TRADD in signaling through tumor necrosis factor receptor 1 and TRIF-dependent toll-like receptors. Nat Immunol.

[93] Varfolomeev E, Maecker H, Sharp D, Lawrence D, Renz M, Vucic D, Ashkenazi A (2005). Molecular determinants of kinase pathway activation by Apo2 ligand/tumor necrosis factor-related apoptosis-inducing ligand. J Biol Chem.

[94] Levin I, Zaretsky M, Aharoni A (2017). Directed evolution of a soluble human DR3 receptor for the inhibition of TL1A induced cytokine secretion. PLoS ONE.

[95] Muck C, Herndler-Brandstetter D, Micutkova L, Grubeck-Loebenstein B, Jansen-Durr P (2010). Two functionally distinct isoforms of TL1A (TNFSF15) generated by differential ectodomain shedding. J Gerontol Biol Sci Med Sci.

[96] Kim S, Zhang L (2005). Identification of naturally secreted soluble form of TL1A, a TNF-like cytokine. J Immunol Methods.

[97] Migone TS, Zhang J, Luo X, Zhuang L, Chen C, Hu B, Hong JS, Perry JW, Chen SF, Zhou JX (2002). TL1A is a TNF-like ligand for DR3 and TR6/DcR3 and functions as a T cell costimulator. Immunity.

[98] Wajant H (2003). Death receptors. Essays Biochem.

[99] Allen Institute for Brain Science. Allen Mouse Brain Atlas [dataset]. Available from mouse.brain-map.org. 2004.

[100] Twohig JP, Roberts MI, Gavalda N, Rees-Taylor EL, Giralt A, Adams D, Brooks SP, Bull MJ, Calder CJ, Cuff S (2010). Age-dependent maintenance of motor control and corticostriatal innervation by death receptor 3. J Neurosci.

[101] Yagita H, Takeda K, Hayakawa Y, Smyth MJ, Okumura K (2004). TRAIL and its receptors as targets for cancer therapy. Cancer Sci.

[102] Dufour F, Rattier T, Shirley S, Picarda G, Constantinescu AA, Morle A, Zakaria AB, Marcion G, Causse S, Szegezdi E (2017). N-glycosylation of mouse TRAIL-R and human TRAIL-R1 enhances TRAIL-induced death. Cell Death Differ.

[103] Wagner KW, Punnoose EA, Januario T, Lawrence DA, Pitti RM, Lancaster K, Lee D, von Goetz M, Yee SF, Totpal K (2007). Death-receptor O-glycosylation controls tumor-cell sensitivity to the proapoptotic ligand Apo2L/TRAIL. Nat Med.

[104] Kichev A, Rousset CI, Baburamani AA, Levison SW, Wood TL, Gressens P, Thornton C, Hagberg H (2014). Tumor necrosis factor-related apoptosis-inducing ligand (TRAIL) signaling and cell death in the immature central nervous system after hypoxia-ischemia and inflammation. J Biol Chem.

[105] Burton TR, Henson ES, Azad MB, Brown M, Eisenstat DD, Gibson SB (2013). BNIP3 acts as transcriptional repressor of death receptor-5 expression and prevents TRAIL-induced cell death in gliomas. Cell Death Dis.

[106] Plissonnier ML, Fauconnet S, Bittard H, Lascombe I (2011). The antidiabetic drug ciglitazone induces high grade bladder cancer cells apoptosis through the up-regulation of TRAIL. PLoS ONE.

[107] Seol DW, Billiar TR (2000). Cysteine 230 modulates tumor necrosis factor-related apoptosis-inducing ligand activity. Cancer Res.

[108] Sheard MA, Asgharzadeh S, Liu Y, Lin TY, Wu HW, Ji L, Groshen S, Lee DA, Seeger RC (2013). Membrane-bound TRAIL supplements natural killer cell cytotoxicity against neuroblastoma cells. J Immunother.

[109] Spierings DC, de Vries EG, Timens W, Groen HJ, Boezen HM, de Jong S (2003). Expression of TRAIL and TRAIL death receptors in stage III non-small cell lung cancer tumors. Clin Cancer Res.

[110] Ehrhardt H, Fulda S, Schmid I, Hiscott J, Debatin KM, Jeremias I (2003). TRAIL induced survival and proliferation in cancer cells resistant towards TRAIL-induced apoptosis mediated by NF-kappaB. Oncogene.

[111] Harper N, Hughes M, MacFarlane M, Cohen GM (2003). Fas-associated death domain protein and caspase-8 are not recruited to the tumor necrosis factor receptor 1 signaling complex during tumor necrosis factor-induced apoptosis. J Biol Chem.

[112] Cui M, Wang L, Liang X, Ma X, Liu Y, Yang M, Liu K, Wei X, Zhou Z, Chen YH, Sun W (2010). Blocking TRAIL-DR5 signaling with soluble DR5 reduces delayed neuronal damage after transient global cerebral ischemia. Neurobiol Dis.

[113] Kamitani T, Nguyen HP, Yeh ET (1997). Activation-induced aggregation and processing of the human Fas antigen. Detection with cytoplasmic domain-specific antibodies. J Biol Chem.

[114] Garcia-Fuster MJ, Ferrer-Alcon M, Miralles A, Garcia-Sevilla JA (2004). Deglycosylation of Fas receptor and chronic morphine treatment up-regulate high molecular mass Fas aggregates in the rat brain. Eur J Pharmacol.

[115] Rossin A, Durivault J, Chakhtoura-Feghali T, Lounnas N, Gagnoux-Palacios L, Hueber AO (2015). Fas palmitoylation by the palmitoyl acyltransferase DHHC7 regulates Fas stability. Cell Death Differ.

[116] Feig C, Tchikov V, Schutze S, Peter ME (2007). Palmitoylation of CD95 facilitates formation of SDS-stable receptor aggregates that initiate apoptosis signaling. EMBO J.

[117] Nishigaki K, Minatoguchi S, Seishima M, Asano K, Noda T, Yasuda N, Sano H, Kumada H, Takemura M, Noma A (1997). Plasma Fas ligand, an inducer of apoptosis, and plasma soluble Fas, an inhibitor of apoptosis, in patients with chronic congestive heart failure. J Am Coll Cardiol.

[118] Abrahams VM, Straszewski SL, Kamsteeg M, Hanczaruk B, Schwartz PE, Rutherford TJ, Mor G (2003). Epithelial ovarian cancer cells secrete functional Fas ligand. Cancer Res.

[119] O’ Reilly LA, Tai L, Lee L, Kruse EA, Grabow S, Fairlie WD, Haynes NM, Tarlinton DM, Zhang JG, Belz GT (2009). Membrane-bound Fas ligand only is essential for Fas-induced apoptosis. Nature.

[120] Tanaka M, Itai T, Adachi M, Nagata S (1998). Downregulation of Fas ligand by shedding. Nat Med.

[121] DeRosa DC, Ryan PJ, Okragly A, Witcher DR, Benschop RJ (2008). Tumor-derived death receptor 6 modulates Nature dendritic cell development. Cancer Immunol Immunother.

[122] Hu Y, Lee X, Shao Z, Apicco D, Huang G, Gong BJ, Pepinsky RB, Mi S (2013). A DR6/p75(NTR) complex is responsible for beta-amyloid-induced cortical neuron death. Cell Death Dis.

[123] Klima M, Zajedova J, Doubravska L, Andera L (2009). Functional analysis of the posttranslational modifications of the death receptor 6. Biochim Biophys Acta.

[124] Kallop DY, Meilandt WJ, Gogineni A, Easley-Neal C, Wu T, Jubb AM, Yaylaoglu M, Shamloo M, Tessier-Lavigne M, Scearce-Levie K, Weimer RM (2014). A death receptor 6-amyloid precursor protein pathway regulates synapse density in the mature CNS but does not contribute to Alzheimer’s disease-related pathophysiology in murine models. J Neurosci.

[125] Luo L, O’Leary DD (2005). Axon retraction and degeneration in development and disease. Annu Rev Neurosci.

[126] Nykjaer A, Willnow TE, Petersen CM (2005). p75NTR–live or let die. Curr Opin Neurobiol.

[127] Roux PP, Barker PA (2002). Neurotrophin signaling through the p75 neurotrophin receptor. Prog Neurobiol.

[128] Bai Y, Li Q, Yang J, Zhou X, Yin X, Zhao D (2008). p75(NTR) activation of NF-kappaB is involved in PrP106-126-induced apoptosis in mouse neuroblastoma cells. Neurosci Res.

[129] Della-Bianca V, Rossi F, Armato U, Dal-Pra I, Costantini C, Perini G, Politi V, Della Valle G (2001). Neurotrophin p75 receptor is involved in neuronal damage by prion peptide-(106–126). J Biol Chem.

[130] Underwood CK, Reid K, May LM, Bartlett PF, Coulson EJ (2008). Palmitoylation of the C-terminal fragment of p75(NTR) regulates death signaling and is required for subsequent cleavage by gamma-secretase. Mol Cell Neurosci.

[131] Monlauzeur L, Breuza L, Le Bivic A (1998). Putative O-glycosylation sites and a membrane anchor are necessary for apical delivery of the human neurotrophin receptor in Caco-2 cells. J Biol Chem.

[132] Ioannou MS, Fahnestock M, ProNGF NGF. Switches from Neurotrophic to Apoptotic Activity in Response to Reductions in TrkA Receptor Levels. Int J Mol Sci. 2017;18:599. 10.3390/ijms18030599 PMC5372615 28282920

[133] Rabizadeh S, Bitler CM, Butcher LL, Bredesen DE (1994). Expression of the low-affinity nerve growth factor receptor enhances beta-amyloid peptide toxicity. Proc Natl Acad Sci U S A.

[134] Sotthibundhu A, Sykes AM, Fox B, Underwood CK, Thangnipon W, Coulson EJ (2008). Beta-amyloid(1–42) induces neuronal death through the p75 neurotrophin receptor. J Neurosci.

[135] Yaar M, Zhai S, Pilch PF, Doyle SM, Eisenhauer PB, Fine RE, Gilchrest BA (1997). Binding of beta-amyloid to the p75 neurotrophin receptor induces apoptosis. A possible mechanism for Alzheimer’s disease. J Clin Invest.

[136] Kim JW, Choi EJ, Joe CO (2000). Activation of death-inducing signaling complex (DISC) by pro-apoptotic C-terminal fragment of RIP. Oncogene.

[137] Lin Y, Devin A, Rodriguez Y, Liu ZG (1999). Cleavage of the death domain kinase RIP by caspase-8 prompts TNF-induced apoptosis. Genes Dev.

[138] McComb S, Shutinoski B, Thurston S, Cessford E, Kumar K, Sad S (2014). Cathepsins limit macrophage necroptosis through cleavage of Rip1 kinase. J Immunol.

[139] Vande Walle L, Wirawan E, Lamkanfi M, Festjens N, Verspurten J, Saelens X, Vanden Berghe T, Vandenabeele P (2010). The mitochondrial serine protease HtrA2/Omi cleaves RIP1 during apoptosis of Ba/F3 cells induced by growth factor withdrawal. Cell Res.

[140] Zhang Y, Spiess E, Groschup MH, Burkle A (2003). Up-regulation of cathepsin B and cathepsin L activities in scrapie-infected mouse Neuro2a cells. J Gen Virol.

