# Supplementary material for: Molecular signatures in prion disease: altered death receptor pathways in a mouse model
Source: J Transl Med. 2024 May 27;22:503. doi: 10.1186/s12967-024-05121-x (PMC11129387; doi:10.1186/s12967-024-05121-x)
Supplement: Supplementary file 1 — Supplementary Material 1 [file 12967_2024_5121_MOESM1_ESM.pdf]

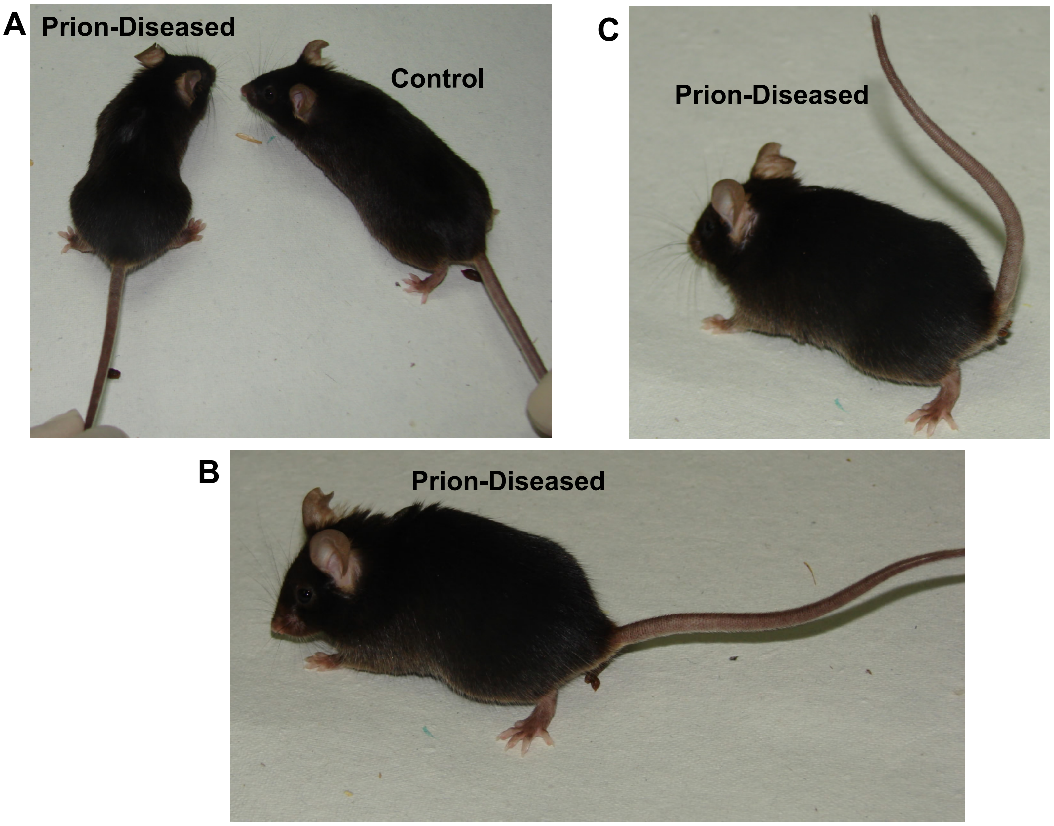

Additional file 1: Fig. S1

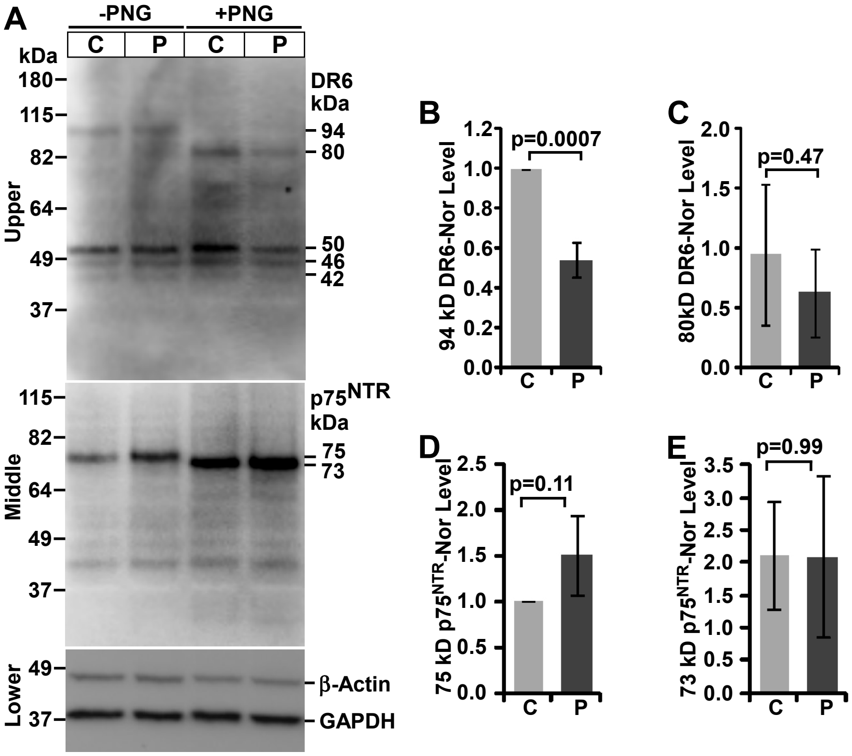

Additional file 1: Fig. S2

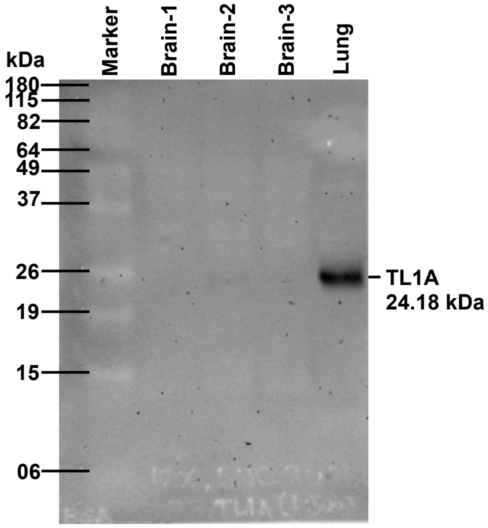

Additional file 1: Fig. S3

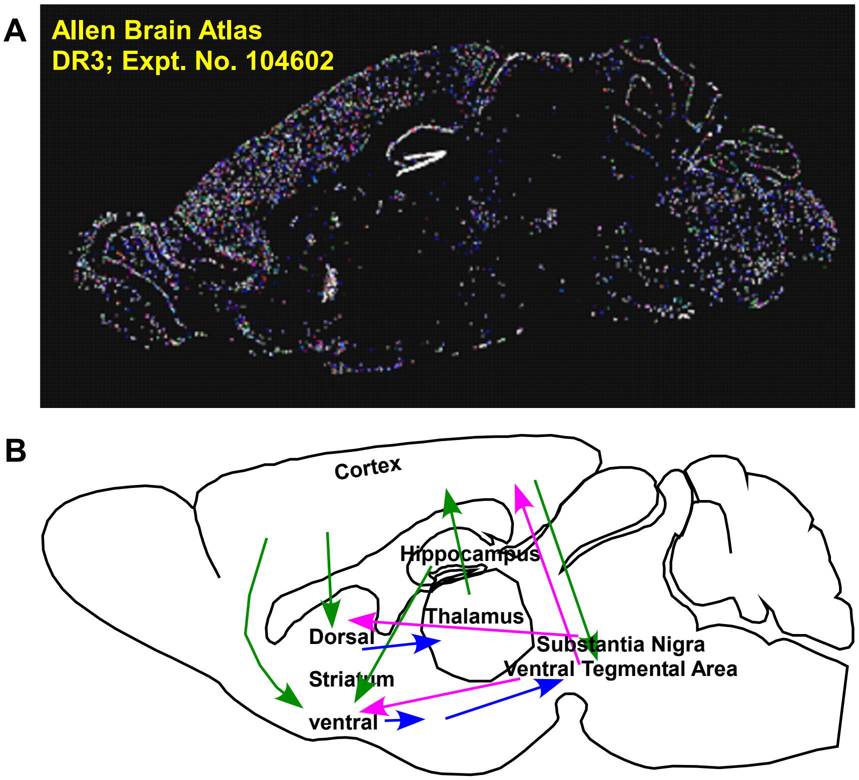

Additional file 1: Fig. S4

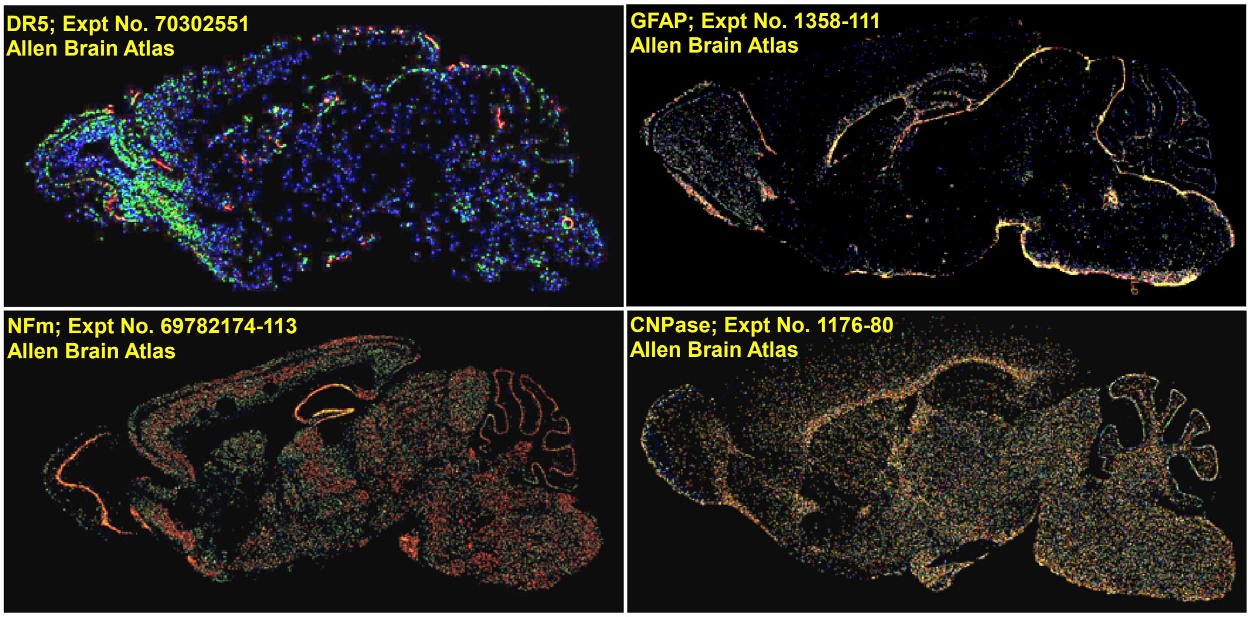

Additional file 1: Fig. S5

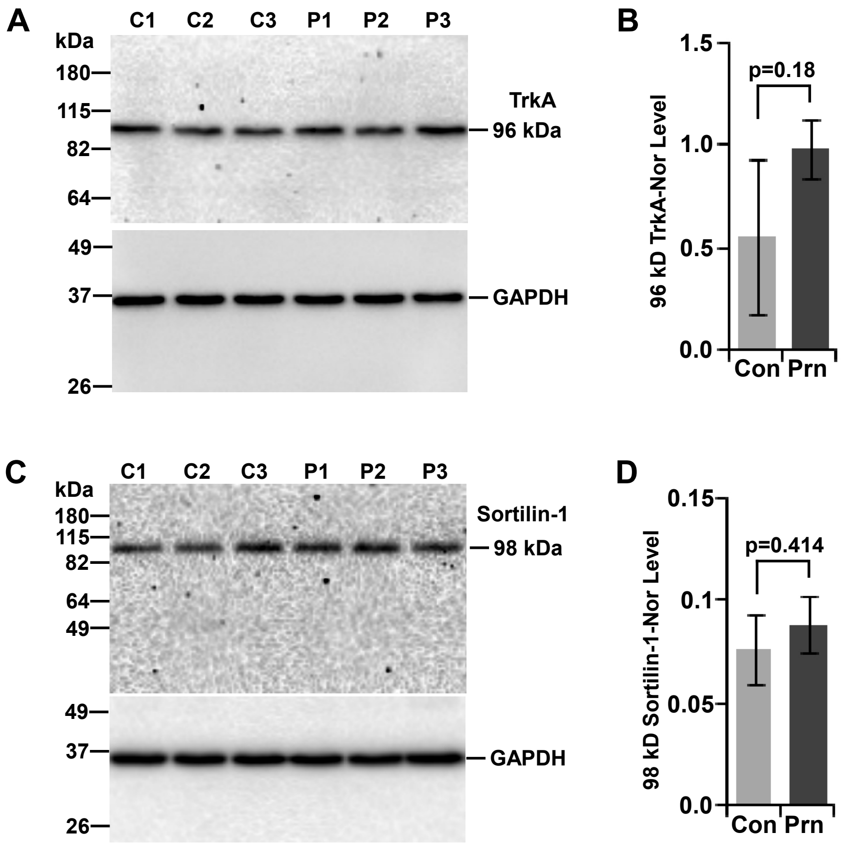

Additional file 1: Fig. S6
