# Supplementary material for: Molecular signatures in prion disease: altered death receptor pathways in a mouse model
Source: J Transl Med. 2024 May 27;22:503. doi: 10.1186/s12967-024-05121-x (PMC11129387; doi:10.1186/s12967-024-05121-x)
Supplement: Supplementary file 9 — Supplementary Material 9 [file 12967_2024_5121_MOESM9_ESM.docx]

**Supplementary Information:**

**Additional file 1**

**Fig. S1** Clinical symptoms of C57BL/6J mice with prion disease. **A** Mouse at the right side is a control mouse, with elongated posture and smooth fur. Mouse at left side is a prion-infected mice clearly exhibiting stiffened body posture with hunchback and wrinkled skin with rough fur. **B** A prion-infected mouse with fixed, wavy tail and stiffened body. **C** when the tail of this prion-infected mouse is lifted upward and released, its tail remained in that position for a while which is uncommon in normal mice. Collectively, prion-infected mice manifested body mass and motor problems typical to various prion diseases.

**Fig. S2** Death receptor (DR6) and p75^NTR^ are N (Asn)-glycosylated. **A** Western blot analysis of brain lysates devoid of SDS post deglycosylation by PNGase F (PNG) show 94-kDa DR6 band shifted to 80 kDa (upper panel) and 75 kDa p75^NTR^ band shifted to 73 kDa (middle panel). Lysates from prion-diseased mice brains (P) show reduced level of 94 kDa and 80 kDa DR6 isoforms than controls (C). GAPDH and β-actin were used as loading controls. **B** Densitometric analysis of 94 kDa DR6 isoform show significant reduced level in prion diseased animals than controls. **C** Densitometric analysis of 80 kDa DR6 isoform show marginal reduction in prion diseased mice brains than controls. **D, E** Densitometry of 75 kDa (D) and 73 kDa (E) p75^NTR^ band show marginal increased expression in prion-diseased mice brain than healthy controls. Each histogram represents mean value of normalized (Nor) intensity ± standard deviation of a set of three control and three prion-diseased mice brains lysates. p≤0.05 is considered statistically significant (two-tailed unpaired *t*-test).

**Fig. S3** Optimization of rat anti-TL1A antibody using three normal C57BL/6J mice brain and one lung lysates. Tissue lysates were prepared in the presence of SDS and denatured by adding β-mercaptoethanol and heating at 97^O^C for 5 mins prior to 12% polyacrylamide gel electrophoresis. Proteins were then transferred onto a 0.2 µm nitrocellulose membrane. Membrane was blocked in TBST containing 5% skimmed milk, followed by immunoblotting with 500-fold diluted anti-TL1A antibody in TBST containing 5% BSA. Membrane was washed three times and incubated with 1:2000-fold diluted HRP-conjugated anti-rat secondary antibody in TBST containing 5% skimmed milk. Membrane was washed three times and incubated with supersignal west pico chemiluminescence reagent (Pierce, Thermo, USA).Time-lapse chemiluminescence images were obtained from 1800 to 3600 seconds. Image shows a prominent band with a molecular mass of approximately 24 kDa from lung lysate but not from any brain lysates. TL1A bands with similar molecular weight were also observed in mouse liver and spleen lysates (data not shown). Therefore, the present TL1A antibody is considered as working.

**Fig. S4** Expression and neuroanatomical significance of DR3 expressing neuronal innervation in normal mouse brain. **A** Comparative *In situ* hybridization analysis of DR3 with respect to GFAP, an established marker of astrocytes, Nefm (neurofilament-m), a marker of neurons, and CNPase (2′,3′-Cyclic-nucleotide 3'-phosphodiesterase), the marker of oligodendrocytes transcripts in normal C57BL/6J mouse brain by Allen Mouse Brain Atlas, mouse.brain-map.org/experiment/show/104602, 1358, 69782174 and 1176 respectively [99]. The Image show DR3 transcripts are expressed in cortex, hippocampus, olfactory lobe, purkinje layer of cerebellum and hindbrain. Expression pattern of DR3 overlaps with neurons and to some extent with oligodendrocytes but not with astrocytes. **B** Innervations from cortex to striatum are glutamatergic in nature and stimulate GABAergic neurons of striatum, which in turn inhibit activation of dopaminergic neurons in substantia nigra and ventral tegmental area. DR3^-/-^ mice have defects in cortico-striatal innervation and results in loss of striatal inhibition of dopaminergic neurons leading to elevated level of dopamine, altered gait, dyskinesia and hyperactivity [100]. Therefore, the increased expression of TL1A, the DR3 ligand along with reduced level of TRADD and TRAF2 in this study, might have killed DR3 expressing cells and exhibit phenotypes similar to DR3^-/-^ mice.

**Fig. S5** A comparative *in situ* hybridization analysis of DR5, (https://mouse.brain-map.org/ experiment/show/70302551) with respect to GFAP, Nefm, and CNPase expression in normal C57BL/6J mouse brain by Allen Mouse Brain Atlas, mouse.brain-map.org/experiment/show/70302551, 1358, 69782174 and 1176 respectively [99]. This meta-analysis shows expression pattern of DR5 gene overlaps mainly with neurons and partly with oligodendrocytes but not with astrocytes. Therefore, decreased expression of DR5 might be due to 1) loss of DR5 expressing neurons and oligodendrocytes to increased expression of TRAIL. 2) It has been reported that expression of DR5 is less in astrocytes but express TRAIL. Expression of DR5 is further reduced in glioblastoma and show resistance towards TRAIL-mediated cytotoxicity [105, 112]. Therefore, reduced DR5 level in this study suggest a novel mechanism towards astrocytosis in prion disease.

**Fig. S6.** Expression of TrkA and sortilin-1 in control and prion-diseased mice brains. **A** Western blot analysis of TrkA expression from three control (C) and three prion-infected (P) mice brain lysates shows a single TrkA band with a molecular mass of 96-kDa in control and diseased mice brain lysates. **B** The densitometry of 96-kDa TrkA protein normalized (Nor) with GAPDH shows mild increased expression of TrkA but without any statistical significance between prion-disease (Prn) mice brains and controls (Con). **C** Western blot analysis of Sortilin-1 protein level shows a single sortilin-1 band with a molecular mass of 98-kDa and without any obvious changes between control (C) and prion-diseased (P) brain lysates. **D** The densitometry of sortilin-1 band normalized with GAPDH shows marginal increase in sortilin-1 level in prion-diseased (Prn) mice brains than controls (Con) but without any statistical significance. Each histogram represents mean value of normalized (Nor) intensity ± standard deviation of a set of three mice brains from control and prion groups. Two-tailed unpaired *t*-test was performed and p<0.05 is considered as statistically significant. Collectively, the involvement of both TrkA and Sortilin found to be insignificant in this mouse prion disease.

**Additional file 2; Legends to supplementary videos**

**Video. S1** This video represents four normal C57BL/6J mice. These normal mice exhibit movement towards exploring their environment in all possible direction but in a controlled manner, interacts with other mice in both the direction in the cage. Occasionally, these mice have the potential to escape the cage in few attempts which are not repetitive in nature. This indicates they have enough strength to grip and full their body upward suggesting normal neuromuscular activities.

**Video. S2** In this video two prion-infected C57BL/6J mice show total loss of normal movement. Both these mice clearly exhibit circling and with restless movement without any specific direction. Their movement is way faster than normal mouse movement. This behaviour represents loss of direction (hippocampus), balance (cerebellum), uncontrolled motor function (cortico-striatal) and possibly heightened fear or aggravation (amygdala).

**Video. S3** This video demonstrates requirement specific movement and friendly interaction among normal mice suggesting adequate healthy brain and muscular function.

**Video. S4** In this video, four prion-infected mice show another unusual phenotype, non-specific movement like repeated and frequent jumping on the inner cage wall than normal mice. This jumping on the wall of the cage can be interpreted as trying to escape, unable to achieve, leading to restlessness. It also suggests weaker fore limb strength or pushing the body upward by hind limb. In both these possibilities, prion mice have profound neuro-muscular problem along with restless and aggravated behaviour.

**Video. S5** In this video, a prion infected mice clearly manifest motor deficit mainly in its hind limb. Towards the end of the video, one can see, the mice could not walk for a while and awkwardly threw the right hind limb with a twisted paw phenotype. This again points towards motor deficits in prion-infected mice.

**Video. S6** In this video, a normal C57BL/6J mouse was tested for his forearm usage using an in-house designed bar. This mouse could able to hold the bar with its claws all through its staying on the bar. It never used its arm pit to restrain from falling suggesting the mouse has powerful fore limb grip. In other videos, the normal mice engage fore and hind limb along with tail to use the bar like a monkey rope, and move towards one end of the bar to escape. These phenotypes speak about the healthy neuromuscular strength along with adequate decision making in normal C57BL/6J mice.

**Video. S7** In this video, a prion-infected mouse when placed on the bar, could not hold the bar with its claws for long, rather used its armpit to stay on the bar for longer, eventually fell down while changing its position. The duration over the bar of this prion-infected mice is way less than control mice. In addition, this infected mouse stayed at the same position and failed to move either side. This clearly suggests prion infected mouse having deficits in neuromuscular strength in both fore limb and hind limb along with deficits in cognition towards escaping the bar by other means as seen in normal mice.
