# Supplementary material for: Molecular signatures in prion disease: altered death receptor pathways in a mouse model
Source: J Transl Med. 2024 May 27;22:503. doi: 10.1186/s12967-024-05121-x (PMC11129387; doi:10.1186/s12967-024-05121-x)
Supplement: Supplementary file 10 — Supplementary Material 10 [file 12967_2024_5121_MOESM10_ESM.pptx]

## Slide 1
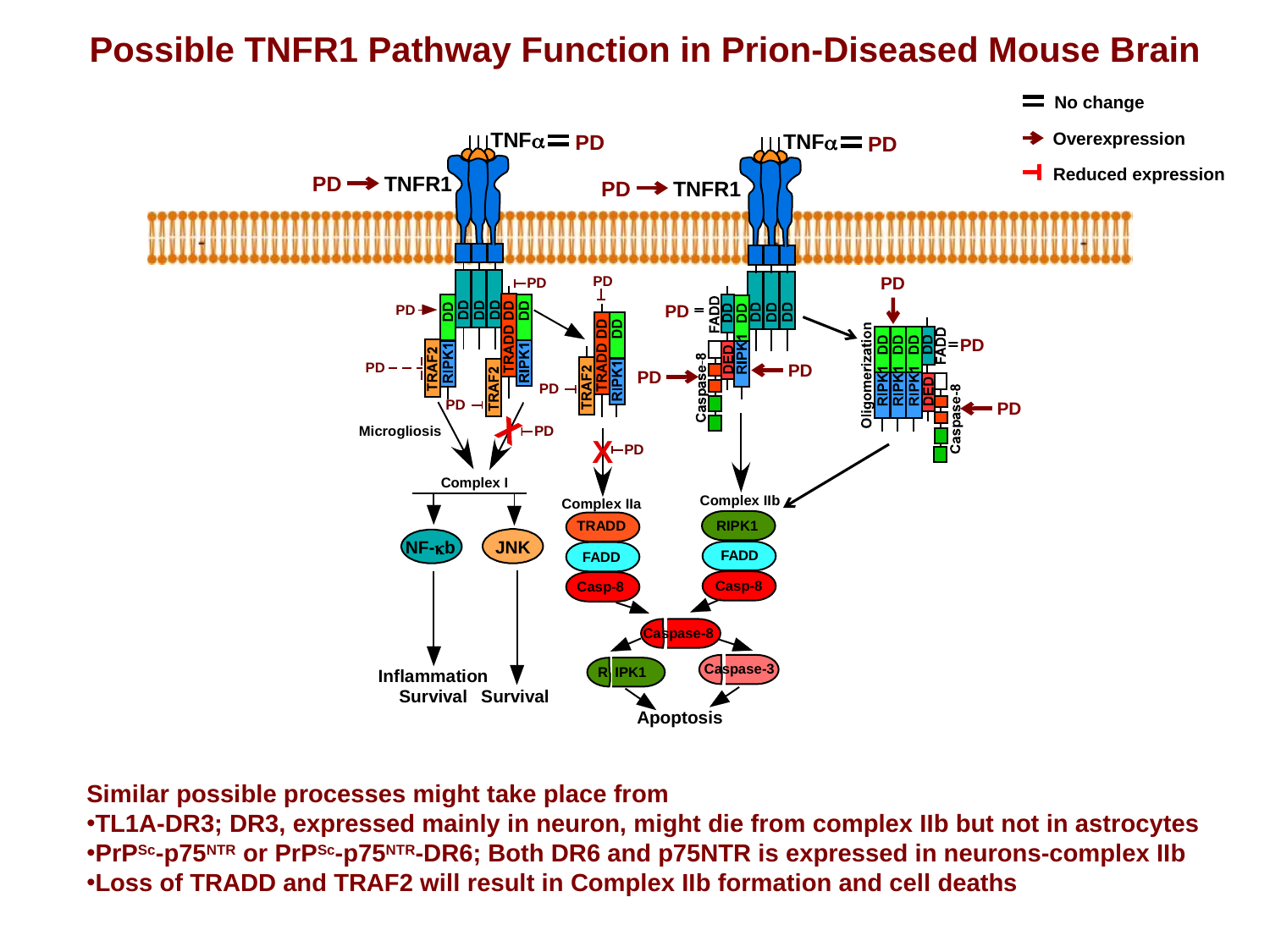

Possible TNFR1 Pathway Function in Prion-Diseased Mouse Brain
No change
Overexpression
Reduced expression
TNFa
PD
TNFa
PD
PD
TNFR1
PD
TNFR1
PD
PD
PD
PD
PD
PD
Similar possible processes might take place from
TL1A-DR3; DR3, expressed mainly in neuron, might die from complex IIb but not in astrocytes
PrPSc-p75NTR or PrPSc-p75NTR-DR6; Both DR6 and p75NTR is expressed in neurons-complex IIb
Loss of TRADD and TRAF2 will result in Complex IIb formation and cell deaths

## Slide 2
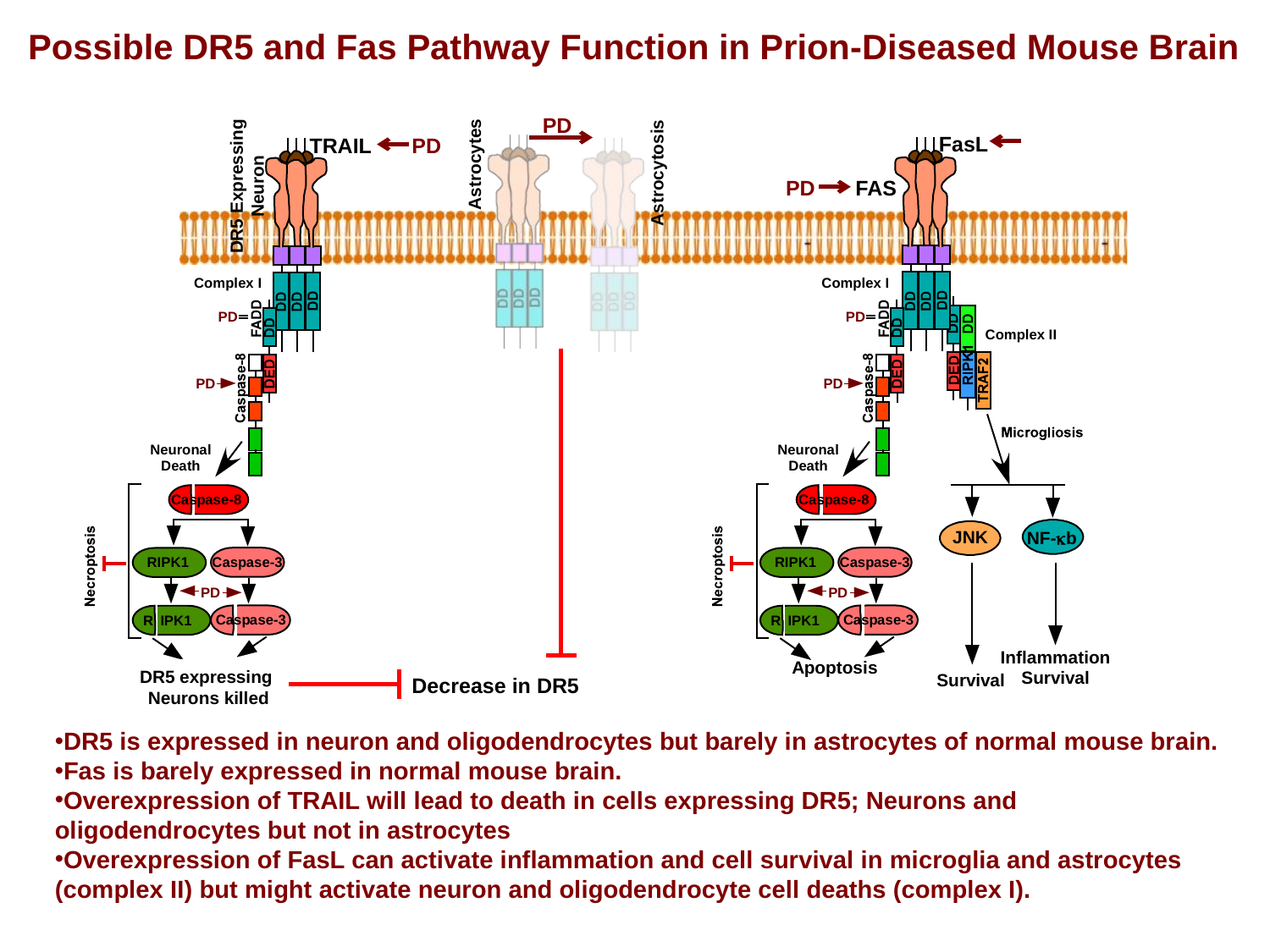

Possible DR5 and Fas Pathway Function in Prion-Diseased Mouse Brain
PD
Astrocytosis
DR5 Expressing
Neuron
Astrocytes
FasL
PD
TRAIL
PD
FAS
Decrease in DR5
DR5 expressing
Neurons killed
DR5 is expressed in neuron and oligodendrocytes but barely in astrocytes of normal mouse brain.
Fas is barely expressed in normal mouse brain.
Overexpression of TRAIL will lead to death in cells expressing DR5; Neurons and oligodendrocytes but not in astrocytes
Overexpression of FasL can activate inflammation and cell survival in microglia and astrocytes (complex II) but might activate neuron and oligodendrocyte cell deaths (complex I).
